# Nutritional and Physiological Effects of Postharvest UV Radiation on Vegetables: A Review

**DOI:** 10.1021/acs.jafc.3c00481

**Published:** 2023-06-22

**Authors:** Frederike Sonntag, Huihui Liu, Susanne Neugart

**Affiliations:** Quality and Sensory of Plant Products, Department of Crop Sciences, University of Göttingen, Carl-Sprengel-Weg 1, 37075 Göttingen, Germany

**Keywords:** vegetables, postharvest UV radiation, phytochemicals, nutritional quality, shelf life

## Abstract

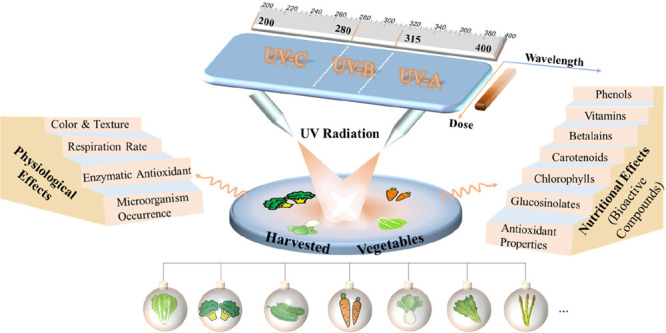

Effective ultraviolet (UV) irradiation has been used as a postharvest technology to reduce decay, delay ripening, and delay senescence in crop products. In this review, the effects of UV radiation of different wavelengths and doses on physiological and phytochemical parameters in postharvest vegetables are discussed in summary, including appearance (color and texture), microbial load, respiration rate, enzymatic antioxidant system, and various bioactive compounds (phenolic compounds, carotenoids, chlorophylls, vitamins, glucosinolates, betalains, and antioxidant activities). In particular, postharvest UV radiation affects oxidative metabolism and increases the antioxidant activity of plant products, which could help delay yellowing and senescence of vegetables, trigger defense responses, and reduce decay and diseases. In some cases, irradiation stimulates the synthesis of bioactive secondary metabolites that may improve the nutritional value of vegetables. The findings presented in this review are very useful and valuable for the preservation and improvement of the nutritional quality of vegetables after harvest. It will also provide scientific support for industrial and commercial applications of UV radiation in postharvest.

## Introduction

Vegetables are rich in phytochemicals, which are considered essential components of a healthy human diet and are beneficial to the human body. Studies have shown that there is a link between a high intake of vegetables and fruits as part of the daily diet and a lower risk of many diseases such as heart disease and stroke.^1−3^ In recent decades, consumer awareness of food quality has been growing and the demand for fresh, nutritious, and minimally processed vegetables rich in health-promoting ingredients has progressively increased. However, freshly harvested vegetables are highly susceptible to qualitative and quantitative deterioration in sensory and nutritional properties and loss due to microbial or enzymatic spoilage.^4^ Preservation technologies and postharvest quality modification have become the focus of research.^5^

Ultraviolet radiation (UV; 100–400 nm, including the UV-C (100–280 nm), UV-B (280–315 nm), and UV-A (315–400 nm) wavebands) comprises a relatively small portion of the total solar radiation. The effects of UV-B and UV-A in plant growth and biochemistry were shown in a number of studies. In this context, there has recently been an increasing number of studies showing the beneficial effects of ultraviolet (UV) light on the postharvest preservation of vegetables. The effects of UV treatment on a particular vegetable product depend on many factors, including the type of UV wavelengths (UV-A, UV-B, or UV-C), intensity, dose, stage of maturity or development of the vegetable, plant species, variety, and uniformity of treatment.^5,6^ Postharvest treatment of vegetables with relatively moderate doses of UV radiation, especially UV-B and UV-C, has been shown to be effective in delaying ripening and senescence, reducing decay, and even improving the quality of vegetables such as broccoli,^7,8^ leafy vegetables,^9−11^ cucumber,^12,13^ tomato,^14^ water bamboo,^15^ bell pepper,^16−18^ etc. Few studies have addressed postharvest UV-A treatment, although it also has the potential to induce phytochemicals and increase the antioxidant activity. In pigeon pea leaves, UV-A was found to have a relatively weak effect on flavonoids and stilbenes compared to UV-B and UV-C radiation.^19^ Different visible wavelengths and doses of UV-A also have an effect on biologically active compounds and antioxidant activity of tomatoes.^20^ Results showed that UV-A treatment at a wavelength of 365 nm had a positive effect on bioactive plant metabolites, such as lycopene and phenolic compounds, and antioxidant activity. With an irradiation duration of at least 180 min, UV-A irradiation was able to achieve a safe effect, enriching photosynthetic pigments, especially carotenoids, activating antioxidant activity, and inducing flavonoids.^20^

However, our understanding of the basic effects of postharvest UV radiation on both the physiology and quality traits of vegetables is still incomplete. A better understanding of the mode of action of UV treatment on the physiological metabolism of vegetables will be helpful in developing more efficient postharvest radiation treatments. Here, we have summarized recent advances in the effect of UV on postharvest vegetable quality and recent applications of UV-A, UV-B, and UV-C in postharvest vegetable storage, including effects on color, various phytonutrients, including phenolic compounds, carotenoids, vitamins, minerals, glucosinolates, and betalanines, and key quality indicators divided into texture, respiration rate, chlorophyll metabolism, and enzymatic antioxidant system. The long-term goal is to develop irradiation protocols. The extent to which extrinsic factors, such as UV radiation itself and its environmental conditions or intrinsic factors originating from the vegetable itself, affect the quality parameters must be examined.

## Appearance and Microbial Decay

### Color

The color and texture of vegetables are decisive factors for the consumer to buy one vegetable or another. UV irradiation of vegetables should have a positive effect on color, especially for red varieties containing anthocyanins, as anthocyanin synthesis can be stimulated by UV radiation. However, at high UV irradiance levels, it is important to check that there are no bleaching effects or texture changes due to water loss caused by radiation-induced heat generation. The aim is that at least if no positive effect can be achieved, ultimately, no change in the desired color and texture will occur.

The inevitable aging of vegetables after harvest is associated with a shorter life span. We have summarized previous studies on the effects of postharvest UV irradiation on color in various vegetables (Table 1). UV-C irradiation can reduce aging symptoms such as visible yellowing in broccoli and cucumber.^12,13^ Reduced postharvest senescence in broccoli by UV-C treatment was explained by reduced chlorophyll degradation at all UV doses (4.0–14.0 kJ m^–2^). However, the highest UV dose resulted in increased phaeophytin content, a degradation product of chlorophyll.^12^ High phaeophytin content is responsible for a color change toward graying. Several processes are responsible for the increase in phaeophytin: for example, membrane disorganization and progressive destruction of thylakoids in the chloroplast. Costa et al.^12^ argue that this is mainly caused by high doses of UV light. The study on cucumber also showed an altered surface of the irradiated side, which hardened without wilting and showed a damaged cuticle layer.^13^ In a study on romaine lettuce, brightness decreased with increasing UV-C dose, in a dose–response relationship starting at the second irradiation dose (12, 24, 36, 54 and 72 kJ m^–2^), while yellow and green colors did not decrease, although this was expected for the green color value at high UV-C doses due to chlorophyll degradation.^21^ The authors argue that higher doses may cause nonenzymatic browning responses. High UV-C doses (40.8 kJ m^–2^) also cause a decrease in brightness and green color and an increase in yellowing in iceberg lettuce during seven days of storage. This is indicative of faster aging and thus loss of the desirable light green color.^22^ In the same study, lower UV-C doses (2.0, 4.1, and 20.4 kJ m^–2^) had no effect on the brightness, green, or yellow color values. Similarly, no change was observed in Chinese cabbage irradiated with a low dose of 1.8 kJ m^–2^.^23^ UV-C treatment may also prevent the increase in green coloration of water bamboo during eight days of storage at 10 °C or two days of storage at 20 °C. The swollen, crisp, white stems of water bamboo, better known as Manchurian wild rice, are consumed raw or cooked after the outer green layer is removed. Color change was measured as brightness, and UV-C irradiation of 4.2 kJ m^–2^ caused the best inhibition of color change (1.1, 2.1, 3.2, 4.2, 5.3, and 6.4 kJ m^–2^).^15^

**Table 1:** Effects of Ultraviolet (UV) Irradiation Treatment on Colors of Vegetables in Postharvest Applied with Different Doses

vegetable	UV wavelength/intensity or dose/distance between UV and plants	storage conditions	measured color parameters	effect	ref
broccoli (*Brassica oleracea* L. var. italica, cv. “Cicco”)	UV-C (254 nm)/4, 7, 10 and 14 kJ m^–2^/30 cm	5 d in the dark at 20 °C	L*, hue	the lightness increased less in irradiated than control, independently of UV-C dose; hue angle reduced and lowest change at 7 kJ m^–2^	(12)
cucumber (*Cucumis sativus* L. cv. “Kanari”)	UV-C (253.7 nm)/3.87, 7.74, and 11.61 kJ m^–2^ (5, 10 and 15 min)/5 cm	10 d in the dark at 15 °C, 60% RH	L*, a*, b*	L* value increased with storage time, but decreased for control; no differences for a* and b*, while a* decreased and b* increased with storage time	(13)
Romaine lettuce (*Lactuca sativa* L. var. longifolia)	UV-C (254 nm)/2 mW cm^–2^ at 12, 24, 36, 54 and 72/8 cm	no storage	L*, a*, b*, net color difference (ΔE*), chroma (C*)	L* was significant reduced from 24 kJ m^–2^; a* was significant decreased but only for 54 kJ m^–2^; b*, C* did not show significant differences; while ΔE* showed no trend with increasing UV-C dose	(21)
iceberg lettuce	UV-C (254 nm)/6.8 mW cm^–2^ at 2.04, 4.08, 20.4 and 40.8 kJ m^–2^/10 cm	0, 1, 3, 5, and 7 d at 4 °C with 57% RH	L*, a*, b*	no significant differences in L*, a*, b* of color up to 5 min (20.40 kJ m^–2^), 10 min with 40.80 kJ m^–2^ dose decreased L* while a* and b* increased over time	(22)
Chinese kale (*Brassica oleracea* var. alboglabra)	UV-C/1.82 kJ m^–2^/25 cm	10 °C for 7 d	L*, b*, hue, chroma	no significant difference in leaf color changes during storage	(23)
water bamboo (*Zizania latifolia*)	UV-C (254 nm)/1.06, 2.12, 3.18, 4.24, 5.30, and 6.36 kJ m^–2^ (2, 4, 6, 8, 10 and 12 min)/30 cm	2 d at 20 ± 2 °C	L*, hue, chroma, and whiteness index	UV-C treatment inhibited an increase in greenness of WBS, control showed green color after 1 d; 4.24 kJ m^–2^ dose maintained better visual appearance than other doses during storage. *L* and whiteness significantly higher at 4.2 kJ m^–2^	(15)
water bamboo (*Zizania latifolia*)	UV-C (254 nm)/4.24 kJ m^–2^(8 min)/30 cm	8 d at 10 ± 2 °C	L*, hue, chroma, and whiteness index	greenness of control was noticeable on day 4 and turning completely green on day 8; L* value and whiteness index were maintained by the UV-C treatment, whereas the control decreased markedly during storage, and the difference was significant after day 4 until the end of storage Hue and chroma values of control markedly increased during storage,	(15)
broccoli (*Brassica oleracea* L. italica) cultivars “Sawayutaka” and “Pixel”	UV-A (342 nm), UV-B (312 nm)/UV-A: 4.5 and 9.0 kJ m^–2^, UV-B: 4.4, 8.8, 13.1, 17.5 and 26.3 kJ m^–2^/15 cm	6 d in the dark at 15 °C	Hue angle value	UV-A treatment did not delay floret yellowing or reduce the hue angle value. 8.8 kJ m^–2^ dose of UV-B displayed more green florets than 4.4 kJ m^–2^ and control. UV-B doses of at least 8.8 kJ m^–2^ significantly delayed decrease of hue angle value. UV-B treatment delayed floret yellowing in both cultivars. “Pixel” florets displayed yellowing more rapidly than “Sawayutaka”	(7)
broccoli (*Brassica oleraceaL. var.* italica cv. “Iron”)	UV-C/10 kJ m^–2^/30 cm	5 d in the dark at 22 °C	hue angle value	samples exposed to UV-C light maintained higher hue values related to controls throughout the storage time period	(24)
broccoli (*Brassica oleracea* L. var. italica “Diplomat”)	UV-B (310 nm)/1.5 and 7.2 kJ m^–2^/20 cm	21 d at 4 °C with 90–95%RH	total color change (Δ*E*)	hormetic dose of 1.5 kJ m^–2^ delayed yellowing of broccoli florets during the storage best compared to the control and stresses at high doses of 7.2 kJ m^–2^	(8)
broccoli (*Brassica oleracea* L. var. italica, cv. “Legacy”)	UV-B (290–340 nm)/3.2, 4.0, and 5.0 W m^–2^ intensity with dose of 2, 4, 8 and 12 kJ m^-2^ (12 treats)/30, 30 and 15 cm	17 d at 4 °C	L*, hue angle	after 17 d hue value decreased and L* increased with the rising doses; the effect of UV-B exposure on color retention is also highly dependent on the irradiation intensity used; at medium and high UV-B intensities the effect on color retention was negligible regardless of the dose applied; the best color results for florets were treated with low doses (2 or 4 kJ m^–2^)	(25)
tomato (*Lycopersicon esculentum* cv. “Zhenfen 202”)	UV-B (311 nm)/10, 20, 40 and 80 kJ m^–2^/20 cm	37 d in the dark at 14 °C with 95% RH	L*, a*, b*	during storage, untreated fruit lost their green color much faster than UV-B treated fruit. UV-B treated fruit, except for the dose of 80 kJ m^–2^, were shinier (higher L* value) than untreated fruit, although the difference was not significant	(26)
yellow bell pepper (*Capsicum annuum* L.)	UV-C/2.2, 4.4, and 6.6 kJ m^–2^ (30, 60, and 90 min)/70 cm	15 d at 12 ± 1 °C	L*, b*	no significant differences were found in both L* and b* between the control and UV-C illuminated samples	(17)
peppers (*Capsicum annum* L. cv. “Zafiro”)	UV-C (254 nm)/7 kJ m^–2^/30 cm	12 and 18 d at 10 °C	L*, hue	After 12 and 18 d of storage, UV-C treated had higher hue values; no differences in L* between control and UV-C treated samples	(18)
tomatoes (*Solanum lycopersicum* L. cv. “Tayfun” F1)	UV-B/0.564 and 1.128 kJ m^–2^/30 cm	7, 14, 21 d at 9 ± 1 °C with 95% RH	L*, a*, hue angle, saturation index	L* and hue angle of tomatoes at red stage were higher compared with other treatments on the 14th d; a* and saturation index of tomatoes was increased during maturation/ripening period; UV-B irradiation accelerated the color development in tomatoes and color development of tomatoes at pink and red harvest stage was delayed by UV-B irradiation	(27)

UV-A and UV-B also have the potential to retard chlorophyll degradation. In a study by Aiamla-or et al.^7^ on broccoli, UV-B doses of at least 8.8 kJ m^–2^ showed the best effect in preventing yellowing of flower heads. At a lower UV-B dose of 4.4 kJ m^–2^ or similar UV-A doses of 4.5 and 9.0 kJ m^–2^, more yellowed flowers occurred during the six days of storage. Similar results were obtained when broccoli was irradiated with a similar UV-C dose and stored in the dark at 22 °C for five days (10 kJ m^–2^).^24^ However, lower UV-B doses may be more suitable for longer storage periods. During 21 days at 4 °C, a lower UV-B dose (1.5 kJ m^–2^) delayed yellowing of broccoli flower heads best compared to the high doses (7.2 kJ m^–2^) or the control.^8^ However, this could also be a cultivar effect, as the cultivar “Diplomat” was used in the study by Duarte-Sierra et al.^8^ In the study by Aiamla-or et al.^7^ the two cultivars were compared and the inflorescences of “Pixel” yellowed faster than those of “Sawayutaka”. Apparently, the applied intensities of UV treatment have a great influence on color. Darré et al.^25^ showed in broccoli that in a short storage experiment of 18 h at 20 °C, no effect of UV treatment could be detected. However, when stored at 4 °C for 21 days, color results were best in flower heads treated with a low dose (2.0 or 4.0 kJ m^–2^). Medium and high doses showed values comparable to the control (up to 12.0 kJ m^–2^).^25^ Ripe green tomatoes also retained their green color better after UV treatment, as untreated fruits lost their green color much faster than UV-B treated fruits (10.0–80.0 kJ m^–2^).^26^ Thus, UV-B treatment may help to extend the storage time. Studies on several bell pepper cultivars showed no color change, regardless of dose (between 2.2 and 7.0 kJ m^–2^) or over extended storage periods of up to 18 days.^16−18^ Tomatoes irradiated with low UV-B (0.6 and 1.1 kJ m^–2^) also showed little effect on color development during 21 days of storage.^27^ UV-A and UV-B radiation is generally higher in the tropics, and tropical fruits may be adapted to higher UV radiation. However, the yellow and red colors of these fruits are carotenoids. No clear trend due to UV radiation in postharvest was identified for carotenoids (see the Carotenoids section). Nevertheless, carotenoids have a protective effect in the presence of excessive light by quenching reactive oxygen species (ROS).^28,29^

These results suggest that high doses of irradiation may accelerate the change to undesirable colors, which are a sign of wilting or spoilage to the consumer. Lower doses have no effect on color parameters or stabilize natural colors over time. Adjusted UV-C doses have the potential to prevent color degradation in green vegetables.

### Texture

The effect of UV radiation on texture, more specifically firmness, has been investigated in only a few studies. Low and medium UV-C doses (2.0, 4.1, and 20.4 kJ m^–2^) had no effect on the firmness of iceberg lettuce during the seven days of storage. Iceberg lettuce treated with the highest UV-C dose (40.8 kJ m^–2^) had lower levels from the first day and was thus softer.^22^ Higher UV-C doses (648 kJ m^-2^) showed the same softening effect as the control in bell peppers during 21 days of storage.^16^ Lower UV-C doses (64.8 and 194.4 kJ m^-2^) showed less softening after 15 days of storage. In two other studies with peppers, medium UV-C doses (6.6 and 7 kJ m^–2^) resulted in a reduction of softening after several days of storage.^17,18^ Similarly, reduced softening was obtained in tomatoes with a low UV-C dose (3.7 kJ m^–2^).^30^ This study also analyzed the cell wall enzymes. The authors demonstrated that UV-C can reduce the activity of cell-wall-degrading enzymes and thus delay softening. Cutting energy and thus toughness of the tissue are other aspects of texture. In white asparagus, the cutting energy of all asparagus spears was increased within four days of storage, and most significantly by UV-C treatment (1.0 kJ m^–2^).^31^ The effect was rather small, possibly due to the low UV-C doses. In another experiment by the same authors, the osmotic potential in white asparagus was also found to be increased by UV-C.^31^ Nevertheless, the result is a consequence of a delayed increase in cell wall components (pectin and lignin) and a greater reduction in cell wall protein in UV-C treated asparagus compared to the control. Reduced lignin formation was also observed in water bamboo after the UV-C treatment. Again, the cutting force of the control increased and remained higher than that of the UV-C treated samples during eight days of storage.^15^

UV-B radiation can also reduce the formation of cellulose and lignin. This was shown in bamboo, where during 15 days of storage, the cellulose content decreased from the third day. Lignin concentration decreased on the sixth day. The strength itself increased in both treated and untreated samples. However, UV-B treatment (8 kJ m^–2^) resulted in lower firmness from day nine compared to the control.^32^ High UV-B doses (20 and 40 kJ m^–2^) have the potential to increase firmness during prolonged storage periods from day 14 until the end of storage. Untreated tomatoes with a very high dose (80 kJ m^–2^) showed the lowest fruit firmness compared with lower doses.^26^

UV-C and UV-B thus have two main effects on fruit softening. They can reduce postharvest hardening by reducing the formation of cellulose and lignin. On the other hand, UV-C and UV-B irradiation can cause vegetables to retain their firmness longer compared to untreated vegetables. This effect is due to a reduction in the activity of cell-wall-degrading enzymes. High doses of UV-C can cause excessive tissue damage, leading to wilting or spoilage. No studies on vegetables are known about the effect of UV-A on the tissue firmness.

### Microbial Load

An important aspect of UV irradiation is to extend the shelf life by reducing the microbial load of food. UV radiation has been shown to cause DNA strand breaks and oxidative damage to lipids in microorganisms and to increase intracellular ROS levels in microorganisms.^33^ Inactivation of pathogens in drinking water and wastewater is already being used commercially.^34,35^ So the question is not whether it is possible to inactivate microorganisms but whether the inactivation is uniform enough for vegetables such as lettuce that have layers and spaces between them. Another consideration is the preservation of desirable quality characteristics, which should not be compromised.

UV irradiation at postharvest has been shown to play an important role in reducing microorganisms on vegetables, such as *Escherichia coli*, *Listeria pseudomonas*, *Salmonella typhimurium*, and *Listeria monocytogenes* (Table 2). Irradiation with UV-C resulted in a reduction of the initial microbial load in almost all cases, as expected.^9,21,22,36^ This reduction was observed in leaves such as spinach,^37,38^ iceberg lettuce,^22,36^ romaine lettuce,^21^ and amaranth,^39^ but also in fruits such as bell peppers^40^ and tomatoes.^36^ Several studies also showed that longer duration and/or higher intensity of UV irradiation had greater efficiency in reducing microbes.^21,22,37,38^ In a few samples, initial irradiation had no effect on microbes: e.g., white asparagus^31^ or iceberg lettuce.^36^ The authors of the study on asparagus used a low dose (1.0 kJ m^–2^) and stated that the initial microbial load was already very low and therefore disinfection was less effective.^31^ Huang and Chen^36^ used a water-assisted UV system for lettuce disinfection (13 or 28 mW cm^–2^) and showed reduction rates comparable to those of other authors. It remains to be assumed that the disinfection effect of UV radiation could be reduced by water but is still effective. Overall, UV radiation has a good disinfection effect on vegetables regardless of whether they have different layers or interstices.

**Table 2:** UV Irradiation in Postharvest Described as Having Roles in Reducing Microbials on Vegetables.

vegetable	UV type (wavelength)/intensity or dose/distance between UV and plants	pathogen	effect	ref
baby spinach (*Spinacia oleracea* L.)	UV-C (254 nm) + water (WUV)/0.2 and 0.3 kJ m^–2^ (2 and 3 min)/17.2 cm	*Listeria monocytogenes*; *Salmonella enterica*	0.2 kJ m^–2^ of WUV was not effective for inactivation of both pathogens; WUV with dose of 0.3 kJ m^–2^ did not improve the efficacy for inactivating *S. enterica*, but enhanced the inactivation of *L. monocytogenes* to 2.0 ± 0.1 log_10_	(9)
iceberg lettuce (*Lactuca sativa* var. capitata)	UV-C (254 nm) + water (WUV) 0.1, 0.3, and 0.5 kJ m^–2^ (1, 3, and 5 min)/17.2 cm	*Listeria monocytogenes*; *Salmonella enterica*	WUV treat effectively inactivated *L. monocytogenes* by 2.1 ± 0.7 log_10_ and *S. enterica* by 2.0 ± 0.6 log_10_ in respect of inoculated populations	(9)
romaine lettuce (*Lactuca sativa* L. var. longifolia)	UV-C (254n m)/2 mW cm^–2^ at 12, 24, 36, 54 and 72 kJ m^–2^/8 cm	*E. coli* NCTC 9001, *S. aureus* NCTC 6571, *S. Enteritidis* NCTC 6676 and *L. innocua* NCTC 11288	UV-C reduced concentrations of all types of microorganisms significantly, but time and bacterial type depended; reduction of all bacteria was achieved after 20 min, finally about 1–1.7 logs at 45 min for all four types	(21)
iceberg lettuce	UV-C (254 nm)/3.40 mW cm^–2^ for 1 min with 50 cm distance at 4 or 25 °C/with distances of 10 or 50 cm at 25 °C	*E. coli* O157:H7, Typhimurium and *L. monocytogenes*	*E. coli* O157:H7, *S. Typhimurium* and *L. monocytogenes* on fresh-cut lettuce were significantly reduced by both treatments but reduction was in all three cases greater at 25 °C/both treatments decreased the three pathogen populations, but 10 cm distance yielded about a 1 log greater reduction	(22)
iceberg lettuce	UV-C (254 nm)/1.36, 2.72, 4.08, or 6.80 mW cm^–2^ for 0.5, 1, 3, 5, or 10 min at 25 °C/10 cm	*E. coli* O157:H7, *S. Typhimurium* and *L. monocytogenes*	higher UV intensity and the longer exposure time inactivation of three pathogen populations the greater the reduction, greatest reduction of over 4 logs after 10 min exposure with 6.80 mW cm^–2^	(22)
iceberg lettuce	UV-C+ water assisted (WUV)/13 or 28 mW cm^–2^ (1 or 2 min)/-	*Salmonella* spp.	reductions of *Salmonella* on lettuce shreds achieved by WUV treatments (1.9 to 2.7 log)	(36)
spinach leaves (*Spinacia oleracea* L.)	UV-A (356 nm)/0.03 mW cm^–2^ at 0.53, 1.07 and 1.60 kJ m^–2^ (30, 60 and 90 min)/13.5 cm	*E. coli* O157:H7, *S. Typhimurium*, and *L. monocytogenes*	UV-A irradiation for 90 min reduced *E. coli* O157:H7, *S. Typhimurium* and *L. monocytogenes* by 0.51, 0.25, and 0.38 log CFU mL^–1^, respectively.	(37)
spinach leaves (*Spinacia oleracea* L.)	UV-C (254 nm)/10 W cm^–2^ at 0.6, 1.8 and 3.0 kJ m^–2^ (1, 3 and 5 min)/18 cm	*L. monocytogenes* and *E. coli* O157:H7	0.6 kJ m^–2^ dose of UV-C reduced *L. monocytogenes*, *E. coli* O157:H7 and pre-existing bacteria by 0.33, 0.66, and 0.67 log CFU g^–1^; 1.8 kJ m^–2^ achieved reduction by 0.97, 1.23, and 1.29 log CFU g^–1^, respectively; 3.0 kJ m^–2^ did not have further reduction	(38)
amaranth (*Amaranthus cruentus* L. cv. “Madiira”)	UV-C (254 nm)/1.7 kJ m^–2^/40 cm	aerobic mesophilic, yeast and mold counts	UV-C significantly lower aerobic mesophilic and yeast counts only at day 0 (harvest day), while after 2 and 4 d, both microbial counts were not significantly different; no significant difference was observed on mold between the UV-C treatment and the control throughout the storage period	(39)
bell pepper (*Capsicum annuum* L. cv “Delphin” and “Bell Boy”)	UV-C (254 nm)/0.22, 0.44, 0.88 or 2.20 kJ m^–2^/-	*Botrytis cinerea*	UV-Ctreatment at all doses reduced fungal decay; infection increased with the dose up to 0.88 kJ m^–2^; a dose of 2.2 kJ m^–2^ caused injury in the form of soft lesions; after 4 weeks storage: nontreated: >90% showed signs of fungal decay, UV-C treatment (0.88 kJ m^–2^): < 0% showed decay sign	(40)
white asparagus (*Asparagus officinalis* L. var. gijnlim)	UV-C (254 nm)/1 kJ m^–2^/-	aerobic mesophilic total bacterial counts, yeast and mold counts	most changes were not significant; a slight inhibition of yeast growth was observed in treated spars especially for combination	(31)
spinach leaves (*Spinacia oleracea* L. cv. “Emilia”)	UV-C/4.54, 7.94 and 11.35 kJ m^–2^/15 cm	mesophilic and psychrophilic aerobic bacteria	after UV-C radiation, the initial microbial load 5.2 log CFU g^–1^ for mesophilic counts was reduced to 4.1–4.9 log CFU g^–1^, then increased after 6 days without marked differences among treatments; similar results were found in psychrophilic bacterial growth throughout the storage	(41)
baby spinach leaves (*Spinacia oleracea* L. cv. “Silver Whale”	UV-C (254.7 nm)/2.4, 7.2, 12 and 24 kJ m^–2^/15 cm	*Listeria*, *Pseudomonas* and *Salmonella*	radiation reduced initial bacterial colonization (d0); no difference after 12 days for *Listeria*, 2 days for *Pseudomonas* and 5 days for *Salmonella*	(42)
lettuce (*Lactuca sativa* L. cv. “Lollo Rosso”)	UV-C (254 nm)/0.407, 0.814, 2.443, 4.07 and 8.14 kJ m^–2^/60 cm	psychrotrophic aerobic bacteria, lactic acid bacteria (LAB), coliform bacteria, yeast and molds	During storage all UV-C doses reduced psychrotrophic bacterial growth; only significant in highest treatments (4.07 and 8.14 kJ m^–2^); UV-C treatments prolonged the shelf life by 3 days based on microbial growth; coliform bacterial growth was significantly inhibited when the highest UV-C radiation dose was applied; however, significant reductions were found at all the UV-C radiation doses from day 0 to 5, similar results were found in yeast; UV-C seemed to stimulate LAB growth and the highest increase was obtained at the highest UV-Cdoses	(11)

The effectiveness of UV-C irradiation in reducing microbes in various vegetables has already been studied in detail. The microbial load after UV-C irradiation during storage was quite variable. In some cases, bacterial and yeast counts were as high as in the control after a few days, e.g. in amaranth^39^ or spinach.^41^ Baby spinach leaves showed no difference from the control in *Pseudomonas marginalis* after two days and in *Salmonella enterica* after five days.^42^ In the same study, *Listeria monocytogenes* contamination was reduced for 12 days.^42^ Bell pepper irradiated with a low dose of UV-C (0.88 kJ m^–2^) were able to be stored much longer due to the reduction in fungal contamination.^40^ Also lettuce of the cultivar Lollo rosso also showed reduced microbial contamination by day eight at higher irradiation doses (4.1 or 8.1 kJ m^–2^).^11^ The authors argue that more microbes are reduced by a lethal UV-C dose at higher irradiation doses. On the other hand, high UV-C doses can also cause tissue lesions. As a result, microbes can easily invade the tissue through the lesions, leading to more rapid deterioration. Therefore, for commercial UV irradiation of vegetables, a range should be selected in which microorganisms are greatly reduced without damaging the tissue.

Few studies were found on the antimicrobial effects of UV-A and UV-B irradiation after harvest. Recently, the combination of UV-A light irradiation with various organic acid treatments has been investigated as a novel antimicrobial treatment for fresh and minimally processed crop products.^37^ UV-A irradiation in combination with acetic acid or phenolic acids such as gallic acid and ferulic acid reduced the inoculation of microorganisms on fresh spinach.^37,43^ UV-A irradiation is able to inactive foodborne pathogens by inducing the formation of free radicals and singlet oxygen with photosensitization by type I and II mechanisms.^44^ However, UV-A cannot inactivate microbes as effectively as UV-C light^45^ and therefore must be supplemented with other antimicrobial treatments to be effective. Combining UV-A irradiation with some food-grade organic acids is a very novel treatment that could have a potential synergistic antimicrobial effect.

### Respiration

High respiration rates of harvested vegetables lead to faster senescence and thus a shortened shelf life. However, not many studies have investigated the relationship among UV radiation, respiration, and ethylene production.

There is no clear trend between UV-C radiation and the respiration rate. Rather, the opposite is true, as all possibilities are shown during storage: increase in lettuce,^10,11^ decrease for carrot, broccoli, asparagus, or pepper,^12,18,31,46^ or no effect of spinach and lettuce.^9,41^ The inconsistency was also found within the lettuce species. In “Lollo rosso” and “Red oak”, increasing respiration rates were observed with increasing UV-C dose (range 0.4–8.1 kJ m^–2^).^10,11^ In contrast, in iceberg lettuce, UV-C irradiation showed no effect (0.1–0.5 kJ m^–2^),^9^ even though the doses were low and it was water-assisted UV-C radiation. UV-C radiation had no effect on the respiration of spinach leaves in two different studies. In the first study, the doses were higher (4.5–11.4 kJ m^–2^) and the period studied was up to 13 days.^41^ In a study by Collazo et al.^9^ the doses were relatively low (0.2 and 0.3 kJ m^–2^) and the storage period was only up to eight days, yet the respiration rate was not affected. Costa et al.^12^ and Vicente et al.^18^ argued that increased respiration is a sign of cellular damage from UV radiation. Thus, if UV-C irradiation caused little or no cellular damage, then respiration would not be altered. Because respiration increased with increasing UV-C dose (1.2–7.1 kJ m^–2^) in a study of “Red oak” lettuce,^10^ this indicates that the hypotheses of Costa et al.^12^ and Vicente et al.^18^ may be correct. However, Allende et al.^10^ argues that UV-C light has the potential to activate several biological processes in higher plants, including stimulation of respiratory activity. Both theories explain very well why increasing effects occur. The lack of effect on iceberg lettuce can also be explained by the low dose (0.1–0.5 kJ m^–2^).^9^ However, this does not explain why respiration decreases in some cases. Huyskens-Keil et al.^47^ found a reduction in the physiological activity of asparagus spears. Also, Costa et al.^12^ argues that lower UV-C doses have the potential to delay tissue damage, thereby reducing reparation for a period of time. Thus, the theory by Costa et al.^12^ and Vicente et al.^18^ may be correct.

There are only two studies investigating the effect of UV-B on respiration, and to our knowledge there are none on UV-A. The respiration rate of broccoli florets increased immediately after UV-B irradiation, but more dramatically at a high dose (1.5 and 7.2 kJ m^–2^).^8^ By day seven, the two UV-B treatments and the control showed almost the same decreased respiration rate of about 180 nmol kg^–1^ s^–1^ CO_2_, which remained constant until the end of storage on day 21. Capia peppers showed no difference between the UV-B treatments (4.5 and 8.9 kJ m^–2^) and control throughout the 49 days of storage.^48^ However, the respiration rate decreased during the first 21 days of storage and then increased. This increase could be a sign of aging. Again, these two studies did not reveal a clear trend but rather showed that UV radiation can cause cellular damage depending on the species.

## Effects on Bioactive Compounds

### Antioxidant Activity Sum Parameters

Sum parameters can usually be analyzed much faster, and the costs for these tests are lower. Therefore, they are a good orientation when certain groups of substances are affected by a stressor. Predominantly, the total phenolic compound (TPC) and total antioxidant capacity (TAC) were analyzed after UV irradiation. In addition, in some cases, the total flavonoid content (TFC) and total carotenoid content (TCC) were analyzed. Phenolic compounds, which include flavonoids, are antioxidants. Among other things, they have a protective function against ROS within the plant cell (see Phenolic Compounds section). Consequently, oxidative stress in plants caused by UV radiation should increase TPC and TFC. TPC and TFC, together with other antioxidants, are part of the TAC, and therefore, TAC should also be increased by UV radiation.

A general positive trend was observed for TPC, TFC, and TAC upon UV irradiation, regardless of the UV wavelength. Increased TPC, TFC, and TAC levels after UV-C irradiation are found in green leafy vegetables, such as in pigeon pea leaves,^19^ amaranth,^49^ and red cabbage^50^ (Table 3). A positive effect of UV-C on TPC, TFC, and TAC was also found in other vegetable forms such as tomato,^51,52^ broccoli,^12,53^ and water bamboo.^15^ Similar positive trends were also detected in pigeon pea leaves,^19^ broccoli,^25,53^ kale,^54^ tomatoes,^20,26^ and carrots^55^ after irradiation with UV-B and UV-A. The results of the studies above indicate that the vegetable form does not play a role in the effect. However, a dependence on extrinsic factors such as dose, cultivation, and UV wavelength was present. A significant difference in TFC between control and UV-C treatment was present on the fourth day for amaranth stored at 20 °C, whereas it was present on the second day at 5 °C.^49^ Thus, lower temperatures may accelerate the increase in the number of flavonoid syntheses. In this experiment, the UV-C doses (1.7 and 3.4 kJ m^–2^) had little effect, as higher doses rarely showed a higher flavonoid concentration. In a study on pigeon pea leaves, UV-C elicited a more pronounced and earlier effect than UV-B and UV-A.^19^ Also, in a study by Dyshlyuk et al.^20^ longer UV-A irradiation times (180 and 360 min) at three different wavelengths (353, 365, and 400 nm) resulted in a stronger increase of TPC and TFC, while a short irradiation for 10 min had no effect. This suggests that a higher dose is required for longer wavelengths to elicit a similar effect. Tomatoes generally appear to tolerate higher UV doses. In a study by Liu et al.^26^ TPC, TFC, and TAC showed increased values during storage for UV-B radiation doses of 20.0 and 40.0 kJ m^–2^ but not for 10.0 and 80.0 kJ m^–2^. However, this correlation was not found in other studies, such as in spinach,^41^ peppers,^18^ and red cabbage.^56^ The UV-C dose for spinach was relatively high (4.5, 7.9, and 11.4 kJ m^–2^), so that TAC and TPC decreased even more drastically.^41^ The authors argue that oxidative stress induced by high UV-C doses probably causes membrane damage and alters the cell composition. This reduced the content of antioxidant compounds. This phenomenon was also detectable in red cabbage with respect to the total anthocyanin content. There was a positive effect at lower UV-C doses (1.0 and 3.0 kJ m^–2^), but not at higher UV-C doses (5.0 kJ m^–2^) until the eighth day of storage. The oxidative stress induced by lower doses leads to a positive feedback loop with increasing levels of TPC, TFC, or TAC. Higher doses have a decreasing effect, as they are likely to lead to increased ROS levels and consequently cellular damage, thus decreasing TPC, TFC, or TAC. In addition, TAC was measured using two different FRAP and ABTS assays on red cabbage cultivar “Zi Guang” and was found to be little affected by increasing UV-C doses (1.0, 3.0, and 5.0 kJ m^–2^) during 12 days of storage.^56^ Nevertheless, the two lower doses in particular were found to increase TAC levels during the first four days of storage. On day 12, both assays were affected differently, possibly a coincidence.^56^ These results indicate that irradiation with intermediate and lower doses has the potential to increase the sum parameters of TPC, TFC, or TAC.

**Table 3:** Effects of Postharvest UV Irradiation on Various Phytochemical Contents of Vegetables

phytochemical				treatment		
categories	compounds measured	effects	plants and UV treatments	UV type (wavelength)/intensity or doses/distance between UV and plants	storage conditions	ref
sum parameters of total antioxidants	total phenolic compound (TPC), total antioxidant capacity (TAC)	all UV triggered TPC and TAC content but intensity verified; UV-C had highest increase; 4 h: no further increase, 8h: severe decline	pigeon pea leaves (*Cajanus cajan* L. Millsp.)	UV-A (365 nm), UV-B (313 nm), UV-C (254 nm)/40 W for 2, 4, 6, and 8 h/50 cm	no storage	(19)
	TPC and TAC	TPC and TAC was increased at both temperature but slightly higher at 4 °C especially toward longer storage	pigeon pea leaves (*Cajanus cajan* L. Millsp.)	UV-B (313 nm)/40 W for 4 h/50 cm	4, 24, 36, 48 and 72 h at 20 or 4 °C	(19)
	Trolox Equivalent Antioxidant Capacity (TEAC)	TEAC was increased 11–39% and 14–17% by UV-C after 2 and 4 d, respectively, but no difference between dose	amaranth (*Amaranthus cruentus* L. cv. Madiira)	UV-C (254 nm)/1.7 and 3.4 kJ m^–2^/40 cm	4 d at 20 °C with 85% RH	(49)
	TAC	3.0 kJ m^–2^ UV-C resulted higher TAC after one day of storage, 1.0 kJ m^–2^ increased TAC but to a lesser extent, nonsignificant decline for 5.0 kJ m^–2^, while after 12 d of storage, 5.0 kJ m^–2^ significantly increased TAC	red cabbage “ZiGuang” (*Brassica oleracea* L. var. capitata f. rubra)	UV-C (253.7 nm)/1.0, 3.0, and 5.0 kJ m^–2^ (50, 150, and 250 s)/-	1, 4, 8, or 12 d in the dark at 4 °C	(50)
	TPC	both untreated and UV-C treated tomato fruit showed increasing trend in TPC; UV-C treated tomato had higher TPC except at 21 days of storage	mature green tomato (*Solanum lycopersicum*, cv. Wanza 15)	UV-C (254 nm)/4 kJ m^–2^/-	7, 14, 21, 28, 35 d in the dark at 13 °C with 95% RH	(51)
	TAC, total phenols and Total flavonoids compound (TFC)	TAC lower in control, only significant in UV-C at day 4 and 6; total phenols increased after UV-C, then displayed lower levels than control after 4 and 6 d of storage; flavonoids also increased during storage both in control and UV-C treated as senescence took place, but lower levels were found in UV-C treated after 4 and 6 d	broccoli (*Brassica oleracea* L. var. italica, cv Cicco)	UV-C (254 nm)/10 kJ m^–2^/30 cm	6 d in the dark at 20 °C	(12)
	TPC and TAC	TPC and TAC were both increased after UV treatments, but no clear trend of dose, intensity or combination	Bimi broccoli	UV-B, UV-B + UV-C/5, 10, and 15 kJ m^-2^ UV-B (9.27 W cm^–2^); UV-B + 9 kJ m^–2^ UV-C (25.21 W cm^–2^)/17.5 cm	24, 48 and 72 h at 15 °C with 90–95% RH	(53)
	total phenols and ascorbic acid (AA), TAC [2,2-diphenyl-1-picryl-hydrazyl-hydrate (DPPH) + ferric reducing ability of plasma (FRAP)]	total phenols of UV-C treated and control shoots remained constant during storage for 2 d and increased afterward, on days 6 and 8, UV-C had higher TPC than control; UV-C had no effect on AA and it decreased on both after 2 d; the antioxidant capacity of both treatments was similar for 4 d, UV-C increased and control decreased after 4 d, both antioxidant capacity of UV-C treated shoots were significantly higher than control on 6 and 8 d	water bamboo (*Zizania latifolia*)	UV-C (254 nm)/4.24 kJ m^–2^ (8 min)/30 cm	8 d at 10 °C	(15)
	TEAC and total phenols	TEAC of control broccoli decreased 12% during storage, none of the 12 different UV-B treatments improved broccoli antioxidant capacity after postharvest storage and rather decreased it; 4.0 and 5.0 W m^–2^ intensity of 12 kJ m^–2^ dose irradiation both maintained similar levels of TEAC after 17 days of refrigerated storage with control	broccoli (*Brassica oleracea* var. italica, cv. Legacy)	UV-B (290–340 nm)/3.2, 4.0, and 5.0 W m^–2^ intensity with dose of 2, 4, 8 and 12 kJ m^–2^ (12 treats)/30, 30 and 15 cm	17 d in the dark at 4 °C	(25)
	TPC and TFC	TPC and TFC increased with UV-B exposure; TPC was not significantly different between treatments while TFC increased with UV-B intensity but no significant difference	kale (*Brassica oleracea* L. var. Acephala)	UV-B/0–3, 3–6 and 6–9 W m^–2^ for 4 h/–	5 d treated 4 h with UV-B stress	(54)
	TPC and TFC	TPC increased at all wavelength at 180 and 360 min irradiances; TFC increased on all cultivars at 365 and 400 nm with 180 min, Bull Heart and Gina increased at 353 nm with 360 min, and all cultivars increased at 180 and 360 min	tomato (*Solanum lycopersicum* L. var. “Budenovka”, “Bull Heart”, and “Gina”)	UV-A (353, 365 and 400 nm)/0.33, 0.28, and 0.28 W m^–2^ (10, 180 and 360 min)/50 cm	36 h at 4 °C	(20)
	TPC, TFC, and TAC	both untreated and UV-B treated fruit showed an increasing trend in TPC during the initial 21 d, then slightly decreased in the following seven days, and again increased toward the end of storage; 20 or 40 kJ m^–2^ UV-B treated tomatoes had significantly higher TFC thane control and other UV-B treatments from day 14 to 28, while at the end of the storage, the differences were not significant; TAC was measured with FRAP and DPPH methods, they were showed similar trends: both untreated and UV-B treated tomatoes showed an increasing trend during the initial 21 d of storage, then slightly decreased over the following seven days, and again increased	mature green tomato (*Lycopersicon esculentum* cv. Zhenfen 202)	UV-B (311 nm)/10, 20, 40 and 80 kJ m^–2^/20 cm	37 d in the dark at 14 °C with 95% RH	(26)
	TPC, TAC, and total carotenoid content (TCC)	TPC and TAC increased in all UV treated samples, carrots chips had the greatest response and baby carrots the lowest; UV treatment did not enhance TCC but a slight decrease for carrot chips	carrot (*Daucus carota* L.)	UV-B/Total dose of 141.4 ± 1.6 mJ cm^–2^ in 14 s with a bimodal peak irradiance of 20.1 ± 0.3 mW cm^–2^/–	3 d at 15 °C with 45% RH	(55)
	TPC and TAC	TAC decreased with time and temperature, but more drastically with higher UV-C application; TPC decrease throughout storage period, especially after 10 d, but not UV-C effect	spinach (*Spinacia oleracea* L. cv. Emilia)	UV-C/4.54, 7.94 and 11.35 kJ m^–2^/15 cm	6, 10 and 13 d at 5 or 8 °C with 80% RH	(41)
	TCC and TAC	no differences for TCC after UV-C treatment, an increased found during storage in both control and UV-C treated, but after 12 and 18 days, UV-C treated displayed lower TCC than control; UV-C increased TAC after treat, while decreased during storage at 10 °C in all fruits, and at 18 d treated fruit showed more TAC than control	pepper (*Capsicum annum* L. cv. Zafiro)	UV-C (254 nm)/7 kJ m^–2^/30 cm	12 and 18 d at 10 °C	(18)
	TFC, TCC, and TAC [2,20-azino-bis(3-ethylbenzothiazoline-6-sulfonic acid) (ABTS) and FRAP]	no clear trend with UV-C on TFC, just general increase until 8 days; UV-C has the negatively effect on TCC with increasing UV-C dose; TAC decreased before 4 days and then increased during the latter period for both the control and UV-C treated in ABTS results; UV-C treatment increased the TAC at 1 day and 4 days, but it decreased lower than the control that was treated with 1 and 5 kJ m^–2^ of treatment in FRAP results	red cabbage (*Brassica oleracea* var. capitata f. rubra cv. “Zi Guang”)	UV-C (254 nm)/1.0, 3.0 and 5.0 kJ m^–2^ (50, 150, and 250 s)/-	1, 4, 8 and 12 d at 4 °C	(56)
	TPC and TCC	all samples decreased significantly in TPC compared to fresh cut; UV samples showed higher TPC during storage than control; TCC decreased considerably and increased at day 7 for UV treated carrots	carrot (*Daucus carota* L. cv. Nantes)	UV-C (254 nm)/0.78 kJ m^–2^/15 cm	3, 5, 7, and 10 d at 5 °C	(46)
	total carotenoid content (TCC), TPC, TFC and TAC	UV-C enhanced TCC content and increased continuously with storage, but no difference just after UV treatment, it showed significantly higher than control after 9 d; TFC content of UV-C treated sample was significantly higher than control at 3 and 6 days, no difference after that; TPC content seemed constant both on UV-C (6.6 kJ m^–2^) treated sample and control during 15 d storage; TAC content was similar on control and treated after UV-C treatment day 0, then it was significantly higher than control, and increased until day 6 and then remained constant for UV-C treated	yellow bell pepper (*Capsicum annuum* L.)	UV-C/2.2, 4.4 and 6.6 kJ m^–2^ (30, 60, and 90 min)/70 cm	15 d at 12 ± 1 °C	(17)
	total phenols, total flavonoids, total flavanol, and TEAC	UV-C radiation induced a significant increase of total phenolics in the peel at MG harvested stage, while flavonoid and flavanol concentration increased in both stages. In the flesh, UV-B treat was effective in increasing flavonoid and flavanol concentration only in MG fruits. UV-B irradiation significantly influenced antioxidant activity in the peel of both genotypes. In the flesh, hp-1 showed a light-dependent increase of antioxidant activity in MG stage fruits	tomato (*Solanum lycopersicum* L.) high pigment-1 (hp-1) mutant and the corresponding wild-type (cv. Money Maker)	UV-B/1.69 W cm^–2^, (1 h, 6.08 kJ m^–2^ per day) until red ripe (RR) stage/45 cm	no storage	(52)
	total carotenoids	the highest value was recorded in the rocket grown under diffused light film with UV-B window and treated with UV-B for 45 s	wild rocket (*Diplotaxis tenuifolia* L.)	UV-B (280–315 nm)/0.2, 0.7, 1.5, and 3.0 kJ m^–2^ for 45, 150, 330 and 660 s, respectively/20 cm	no storage	(57)
vitamins	vitamin C (Vc)	multiple UV-C treatments indicated a great deal of enhancement in Vc content, which almost made this content remain at the initial level, by contrast, Vc contents in control and single UV-C treated leeks dropped rapidly to less than 0.02 mg/g.	leek	UV-C (254 nm)/2.46 kJ m^–2^ for 5 min (single or once every day for multiple)/20 cm	1, 2, 3, 4, 5 d at 4 °C	(60)
		a small decrease in Vc content treated by multiple UV-C treatment, while the contents in both control and single UV-C treated spinaches declined sharply on day 1, afterward, this content decreased to the initial level on day 3, and finally remained there.	spinach	UV-C (254 nm)/2.46 kJ m^–2^ for 5 min (single or once every day for multiple)/20 cm	1, 2, 3, 4, 5 d at 4 °C	(60)
		multiple UV-C irradiations had improved Vc content by day 1; for control and single UV-C treated cabbages, their Vc contents decline rapidly, but Vc content of the single UV-C treated was always higher than control	cabbage	UV-C (254 nm)/2.46 kJ m^–2^ for 5 min (single or once every day for multiple)/20 cm	1, 2, 3, 4, 5 d at 4 °C	(60)
	ascorbic acid (AA)	influence of UV-C was week, it was significantly reduced by 5 min of UV-C at 3 d and increased at 10 d, which also competed with water loss	cucumber (*Cucumis sativus* L., cv. Kanari)	UV-C (254 nm)/12.9 W m^–2^ with 0, 5, 10 and 15 min/5 cm	10 d in the dark at 15 °C with 60% RH	(13)
	AA	UV-C treated pepper showed slightly in AA content than control, no significant difference	yellow bell pepper (*Capsicum annuum* L.)	UV-C/2.2, 4.4 and 6.6 kJ m^–2^ (30, 60, and 90 min)/70 cm	15 d at 12 ± 1 °C	(17)
	AA	during the first week of storage, AA content increased slightly in both control and UV-C treated pepper, 5% increase observed during the first 7 days of storage, later on, no variations in AA were detected in UV-C treated fruits, while a reduction was found in the control after 21 d of storage	peppers (*Capsicum annum* L. cv Cornago)	UV-C (254 nm)/10 kJ m^–2^/30 cm	7, 14 and 21 d at 0 °C	(61)
	AA	AA did not change after UV-A or UV-B irradiation, 3 days of irradiation with UV did not show adverse changes in AA contents compared to nonirradiated	parsley (*Petroselinum crispum* Mill.)	UV-A and UV-B/3 days of radiation; UV-A or UV-B for 5 min at 98 μmol m^–2^ s^–1^/-	6 d at 10 °C with 60% RH	(63)
	AA	UV-B treated and untreated tomato all showed an increasing trend in AA content during the whole storage period, however, UV-B had a negative effect on AA content during storage; At the end of storage, the average AA contents of UV-B treated fruit were about 18% lower than those of untreated fruit	mature green tomato (*Lycopersicon esculentum* cv. Zhenfen 202)	UV-B (311 nm)/10, 20, 40 and 80 kJ m^–2^ (6.0 ± 0.1 W m^–2^)/20 cm	37 d in the dark at 14 °C with 95% RH	(26)
	total AA (sum of reduced and oxidized forms)	total AA content was positively affected by UV-B treatment in both skin and flesh of MM and at any harvesting stage; No significant change was observed in hp-1 peel while in the flesh of mature green tomatoes AA concentration slightly decreased in comparison to control	tomato [*Solanum lycopersicum* L. cv. high pigment-1 (hp-1) mutant and wild-type “Money Maker” (MM)]	UV-B/1 h, 6.08 kJ m^–2^ d^–1^ until red ripe stage/45 cm	18 °C with 80% RH	(64)
	vitamin E	UV-C did not affect vitamin E content during storage, except after 14 d where leaves treated with UV-C had higher vitamin E content; both UV-C treatments did not differ with each other	amaranth (*Amaranthus cruentus* L. cv. Madiira)	UV-C (254 nm)/1.7 and 3.4 kJ m^–2^/40 cm	2, 4 d at 20 °C and 2, 4, 14 d at 5 °C with 85% RH	(49)
phenolic compound	7 flavonoids: naringenin, luteolin, apigenin, orientin, pinostrobin chalcone, pinostrobin and apigenin-6,8-di*C*-α-l-arabinopyranoside (F1, F2, F3, F4, F5, F6 and F7); 2 stilbenes: longistyline C and cajaninstilbene acid (S1 and S2)	UV-C: the longer the irradiation tested time, the higher F1, F2 and F3 content; other compounds showed trend of an increase first then a decline; UV-B: F4, F5 and F6, S1 and S2 had similar trend as induced by UV-C, no significant changes with F1, F2, F3 and F7; UV-A: similar to UV-B, the changes were less marked	pigeon pea leaves (*Cajanus cajan* L. Millsp.)	UV-A (365 nm), UV-B (313 nm), UV-C (254 nm)/40 W for 2, 4, 6, and 8 h/50 cm	no storage	(19)
		no clear change for F4 and F7 at both of 20 and 4 °C; F5, F6, S1 and S2 increased at both temperatures; difference between 20 and 4 °C: F1, F2 and F3 increased with time at 20 °C	pigeon pea leaves (*Cajanus cajan* L. Millsp.)	UV-B (313 nm)/40 W for 4 h/50 cm	4, 24, 36, 48, and 72 h at 20 or 4 °C	(19)
	3 major peaks: Cy3diG5G, Cy3*p*CdiG5G, Cy3(si)diG5G; 12 minor peaks	here found 11 and 15 anthocyanins on control and UV-C treated samples; Cy-3-*O*-glucoside-5-*O*-glucoside (P2), Cy-3-*O*-(feruloyl)-glucoside-*5-*O*-*glucoside (P11) and Cy-3-*O*-(sinapoyl)-glucoside-5-*O*-glucoside (P12) increased with rising UV-C dose; higher UV-C dose decreased accumulation of Cy3diG5G, Cy-3-*O*-(feruloyl)-triglucoside-5-*O*-glucoside), Cy-3-*O*-(sinapoyl)-triglucoside-*5-*O*-*glucoside and Cy3(si)diG5G during storage; 3.0 kJ m^–2^accumulates highest sum of anthocyanins at 8 d of storage	red cabbage “ZiGuang” (*Brassica oleracea* L. var. capitata f. rubra)	UV-C (253.7 nm)/1.0, 3.0, and 5.0 kJ m^–2^ (50, 150, and 250 s)/–	1, 4, 8, or 12 d in the dark at 4 °C	(50)
	6-methoxymellein (6-m)	UV dose of 2.20 × 10^5^ erg cm^–2^ was found to be the most effective and induced an accumulation of 6-m; 6-m accumulation in UV-treated carrot slices reached a maximum within 48 h at room temperature	carrot (*Daucus carota* L. cv. Caropak or Navajo	UV-C (254 nm)/2.20 × 10^5^ erg cm^–2^/-	48, 96 h in the dark at 20 °C	(67)
	6-methoxymellein	UV-C irradiation was able to significantly increase 6-m level in carrots; after 5, 10 and 20 d of UV treat, highest 6-m accumulation was in whole surface carrot followed by crown carrot and lower half of carrot	carrot (cv. Carson)	UV-C (254 nm)/0.88 kJ m^–2^/40 cm	5, 10, and 20 d at 10 °C with 90% RH	(68)
	*p*-coumaric acid, cafferic acid, trans-ferulic acid, chlorogenic acid, gallic acid, protocatechuic acid, rutin, naringenin, and quercetin	UV-C significantly stimulated the accumulation of individual phenolic acids and flavonoids (PA&F) after 21 days and 28 days of storage except for naringenin, however, UV-C irradiation did not affect the content of chlorogenic acid, gallic acid, rutin and quercetin at the end of the storage period; all individual PA&F increased in the early storage and then decreased further	mature green tomato (*Solanum lycopersicum*, cv. Wanza 15)	UV-C (254 nm)/4 kJ m^–2^/-	7, 14, 21, 28, or 35 in the dark at 13 °C at 95% rH	(51)
	quercetin, kaempferol derivatives and total flavonoids	flavonoid contents (quercetin and kaempferol derivatives, as well as total flavonoids) increased within the storage period and depending on the UV-C irradiation dose and storage temperature	amaranth (*Amaranthus cruentus* L. cv. Madiira)	UV-C (254 nm)/1.7 and 3.4 kJ m^–2^/40 cm	4 d at 20 °C with 85% RH	(49)
	*p*-coumaric acid, jaceidin, kaempherol glycoside, apigenin aglycone, vitexin, isovitexin	the content of *p*-coumaric acid increased, and jaceidin was newly produced in spinach leaves by 3 days of treat with UV-B during 6 days storage; both UV-A and UV-B slightly and significantly increased kaempherol glycoside content in radish sprouts on 3 days of storage and maintained this higher value on 6 d compared to control; in parsley, the aglycone form of apigenin significantly increased by 7-fold with UV-B and by 2.5-fold with UV-A compared to control, and its glycoside forms significantly accumulated in plants irradiated with UV-B on 6 days; vitexin content in Indian spinach significantly increased on 3 days in UV-B radiation and on 6 days with both UV lights compared to the content before storage; a minor flavonoid, kaempherol glycoside, also increased on the sixth day by both UV-B and UV-A, and isovitexin increased by UV-A	spinach (*Spinacia oleracea* L.), radish (*Raphanus sativum* L.), parsley (*Petroselinum crispum* Mill.), Indian spinach (*Basella rubra* L.), garden pea sprout (*Pisum stivum* L.), and watercress (*Nasturtium officinale* R. Br.)	UV-A and UV-B/3 days of radiation; UV-A or UV-B for 5 min at 98 μmol m^–2^ s^–1^	6 d at 10 °C with 60% RH	(63)
	hydroxycinnamic acids (HCAs)	1.5 and 7.2 kJ m^–2^ of UV-B enhanced the titer of total HCA by 12%; the content of 1-sinapoyl-2-feruloyl gentibiose were significantly increased by both doses of UV-B	broccoli (*Brassica oleracea* L. var. italica “Diplomat”)	UV-B (310 nm)/1.5 and 7.2 kJ m^–2^/20 cm	4, 7 and 14 d at 4 °C with 90–95% RH	(8)
	5-*O*-caffeoylquinic acid (5-CQA) or/and chlorogenic acid	5-CQA and chlorogenic acid was increased in all UV treated samples; carrot chips were the most responsive and baby carrots were the least responsive	carrot (*Daucus carota* L.)	UV-B/Total dose of 141.4 ± 1.6 mJ cm^–2^ in 14 s with a bimodal peak irradiance of 20.1 ± 0.3 mW cm^–2^/–	3 d at 15 °C with 45% RH	(55)
	total hydroxycinnamic acid; coumaroyl glycoside	hydroxycinnamic acid contents were low before treatment; coumaroyl glycoside was not present in fresh leaves as well as after storage in the dark (only trace amounts partial), but found in UV-B treated leaves in dramatically high amounts, up to 1 mg g^–1^ dm in the 4 days stored samples under UV-B	white cabbage var. Lennox	UV-B (290–315 nm)/0.3–0.4 W m^–2^, 12 h per day/–	2, 4 and 7 d at 4 °C	(75)
	quercetin, kaempferol, and isorhamnetin	significantly higher contents of flavanols in flower buds, than at harvest or after prestorage, were found in broccoli stored at 10 °C and exposed to visible light and UV-B irradiation; radiation treatments during storage significantly affected the content of quercetin, for which the combination of visible light and UV-B radiation produced higher contents than the lowest visible light level	broccoli (*B. oleracea* L. var. italica, cv. Marathon)	UV-B (280–315 nm)/0.23 W m^–2^, 12 h per day/–	prestorage in the dark at 0 or 4 °C, then 3 days storage at 10 or 18 °C	(69)
	flavanol index with Dualex plus sensor, total flavanols as sum of quercetin, kaempferol, and their glycoside	during storage, no significant changes in FLAV were observed on the apical part of both UV treated and untreated leaves. Instead, a marked increase in FLAV was found on the basal adaxial sides of leaves; The flavanol content of cabbage leaves after 3 days of storage rose to 4.61 mg g^–1^ in the UV-B lamp irradiated samples	white cabbage (*Brassica oleracea* L. var. capitata subvar. alba),	UV-B (313 nm)/0.34 and 0.43 W m^–2^ (12 h per day for three days)/140 cm	no storage	(70)
	HCA	In UV-B treated peel, *p*-coumaric acid (CA) showed double concentration of control, in MG fruits, leading to about 196- and 3.4-fold increase of caffeic and ferulic acid, but no accumulation for sinapic acid (SA). Instead, MM fruits harvested at TU stage showed a decrease of caffeic and ferulic acid in the peel, while SA did not change. In MM flesh, higher amount of caffeic and SA was measured after UV-B treat, while ferulic acid underwent a slight but significant increase in MG fruits and a decrease in TU ones, and CA was unaffected. UV-B treatment increased caffeic, ferulic and CA of hp-1 peel at MG, being ineffective (ferulic and CA) or leading to a decrease on caffeic acid in the peel of TU fruits. At the flesh level, only caffeic and ferulic acids of hp-1 fruits were significantly increased at MG stage	tomato (*Solanum lycopersicum* L.) high pigment-1 (hp-1) mutant and the corresponding wild-type (cv Money Maker)	UV-B/1.69 W cm-2, (1 h, 6.08 kJ m^–2^ per day) until red ripe (RR) stage/45 cm	no storage	(52)
carotenoids	lycopene, β-carotene, and lutein	UV-B enhanced β-carotene, lycopene and lutein concentration in MM peel, independently form the harvesting stage, while only lycopne was enhanced in the flesh of the cultivar MM, Hp-1 mutant was characterized higher carotenoid concentration than MM, did not experience any influence of UVB, modest decrease in flesh lutein concentration, in particular in mature green fruits	tomato (*Solanum lycopersicum* L.) high pigment-1 (hp-1) mutant and wild-type (“Money Maker”)	UV-B/1 h, 6.08 kJ m^–2^ d^–1^ until red ripe stage/45 cm	18 °C with 80% RH	(64)
	lycopene, β-carotene, and lutein	after UV-A treatment, the content of β-carotene, lycopene, lutein increased in the samples, while the indicator reached its peak at a wavelength of 365 nm	tomato (*Solanum lycopersicum* L. var. “Budenovka”, “Bull Heart” and “Gina”)	UV-A (353, 365 and 400 nm)/0.33, 0.28, and 0.28 W m^–2^ (10, 180 and 360 min)/50 cm	36 h at 4 °C	(20)
	total carotenoids, xanthophyll/ carotene ratio	amaranth leaves showed a strong decline in carotenoids during storage at 20 °C, the decline was retarded by higher UV-C dosas of 3.4 kJ m^–2^, total carotenoid contents were significantly lower in leaves of both UV-C treatments until 2 days, thereafter (10 and 14 days), contents of both pigments were significantly higher with increasing UV-C doses	amaranth (*Amaranthus cruentus* L. cv. Madiira)	UV-C (254 nm)/1.7 and 3.4 kJ m^–2^/40 cm	4 d at 20 °C and 14 d at 5 °C with 85% RH	(39)
	lycopene, β-carotene, and lutein	all 3 carotenoids decreased after storage both at 20 and 5 °C; At 20 °C, 3.4 kJ m^–2^ resulted in higher β-carotene and lutein after 2 d of storage, while no difference found with 1.7 kJ m^–2^; after 4 days of storage, higher lutein content with increasing UV-C doses; at 5 °C, after 4 days of storage, the reduction was still observed for 1.7 kJ m^–2^ UV-C treated leaves in all three carotenoids, whereas no difference was observed with 3.4 kJ m^–2^, after 14 days of storage, both UV-C treatments resulted in higher three carotenoids with increasing dose	amaranth (*Amaranthus cruentus* L. cv. Madiira)	UV-C (254 nm)/1.7 and 3.4 kJ m^–2^/40 cm	2, 4 d at 20 °C and 2, 4, 14 d at 5 °C with 85% RH	(49)
chlorophyll	chlorophyll a and b (Chl a and Chl b), pheophytin (Pheo)	chlorophyll a and b decreased during storage at 20 °C, UV-C delayed chlorophylls degradation; an accumulation was observed after 2 and 4 days at 20 °C, followed by a reduction after 6 d; no differences in Pheo content after UV-C treat and also after 2 days, but afterward accumulated less Pheo than control	broccoli (*Brassica oleracea* L. var. italica, cv Cicco)	UV-C (254 nm)/10 kJ m^–2^/30 cm	6 d in the dark at 20 °C	(12)
	total chlorophyll contents	UV-C samples maintained higher total chlorophyll level related to controls throughout the storage time	broccoli (*Brassica oleracea* L. var. italica)	UV-C/10 kJ m^–2^/30 cm	5 d in the dark at 22 °C	(24)
	Chl a, Chl b, and total chlorophylls	UV-C delayed both chlorophylls degradation, the general trend was to maintain the initial chlorophyll content during shelf life.	spinach (*Spinacia oleracea* L. cv. Emilia)	UV-C/4.54, 7.94 and 11.35 kJ m^–2^/15 cm	6, 10 and 13 d at 5 or 8 °C with 80% RH	(41)
	Chl a	Chl a of multiple UV-C treated leeks had declined a little to about 0.3 mg g^–1^ by day 2, this content then remained there	leek	UV-C (254 nm)/2.46 kJ m^–2^ for 5 min (single or once every day for multiple)/20 cm	1, 2, 3, 4, 5 d at 4 °C	(60)
	Chl a	Chl a of multiple UV-C treated spinach decreased only slightly during 5 days storage, while those of control and single UV-C treated both declined rapidly and simultaneously to only 0.15 mg/g	spinach	UV-C (254 nm)/2.46 kJ m^–2^ for 5 min (single or once every day for multiple)/20 cm	1, 2, 3, 4, 5 d at 4 °C	(60)
	Chl a	Chlorophyll a of multiple UV-C treated cabbages was significantly higher than those of control and single UV-C treated cabbages, which only decreased slightly	cabbage	UV-C (254 nm)/2.46 kJ m^–2^ for 5 min (single or once every day for multiple)/20 cm	1, 2, 3, 4, 5 d at 4 °C	(60)
	Chl a, Chl b, and total chlorophyll contents	Amaranth leaves showed a strong decline in chlorophyll during storage at 20 °C, which was not inhibited by any of the UV-C; At 5 °C, amaranth had significantly lower chl a and b contents in both UV-C treatments within 4 days, while after 10 and 14 days, chl a was significantly higher with the increase in UV-C dose and chl b was significantly higher at 1.7 kJ m^–2^ compared to the control	amaranth (*Amaranthus cruentus* L. cv. Madiira)	UV-C (254 nm)/1.7 and 3.4 kJ m^–2^/40 cm	4 d at 20 °C and 14 days at 5 °C with 85% RH	(39)
	total chlorophyll	UV-C increased total chlorophyll but no intensity variation at 0 days; while it was reduced for irradiation from 3 to 10 days	cucumber (*Cucumis sativus* L., cv. Kanari)	UV-C (254 nm)/12.9 W m^–2^ with 0, 5, 10 and 15 min/5 cm	10 d in the dark at 15 °C with 60% RH	(13)
	Chl a, Chl b and total chlorophyll contents	Chl a, Chl b and total chlorophyll contents of control were significantly higher than UV-C treated shoots, 4.24 kJ m^–2^ UV-C effectively inhibited chlorophyll biosynthesis of water bamboo	water bamboo (*Zizania latifolia*)	UV-C (254 nm)/4.24 kJ m^–2^/30 cm	8 d at 10 °C	(15)
	total chlorophyll content	UV-B treatment delayed floret yellowing in both cultivars. “Pixel” florets displayed yellowing more rapidly than “Sawayutaka” cultivar florets; Chl contents were significantly higher in “Sawayutaka” with UV-B treatment as compared to “Pixel” with UV-B treatment on day 6; These results indicated that “Sawayutaka” responded more strongly to UV-B treatment than “Pixel”.	broccoli (*Brassica oleracea* L. italica cv. “Sawayutaka” and “Pixel”)	UV-B (312 nm)/8.8 kJ m^–2^/15 cm	6 days in the dark at 15 °C	(7)
	Chl a and Chl b	Chl a and b contents in the control showed a slight decrease forthe first 2 days of storage at 15 °C and then decreased rapidly concomitant with floret yellowing; the effect of UV-B treatment on Chl degradation was revealed on day 3, UV-B treatment suppressed the decline in Chl level during storage and delayed the progress of floret yellowing by around 2 days	broccoli (*Brassica oleracea* L. italica, cv. Sawayutaka)	UV-B (312 nm)/19 kJ m^–2^/15 cm	36 h at 4 °C	(80)
	Chl a and Chl b	for all three different films, the UV-B treatment for 45 s elicited higher values of chlorophyll a and was different from the other treatments of each film; both UV-B treatments for 45 and 150 s, elicited the highest values, which were significantly different from all other treatments	wild rocket (*Diplotaxis tenuifolia* L.)	UV-B (280–315 nm)/0.2, 0.7, 1.5, and 3.0 kJ m^–2^ for 45, 150, 330 and 660 s respectively/20 cm	no storage	(57)
glucosinolates (GLs)	glucoraphanin, glucobrassicin, neoglucobrassicin, 4-hydroxyglucobrassicin and 4-methoxyglucobrassicin (G1, G2, G3, G4, and G5)	1.5 and 7.2 kJ m^–2^ doses of UV-B increased the total glucobrassicins by 18%, and 22%; G2 was enhanced by 15% in both UV-B doses group; G1 increased by 11% and 16% in florets with 1.5 and 7.2 kJ m^–2^ UV treated respectively; G3, G4, and G5 were also increased but not significant	broccoli (*Brassica oleracea* L. var. italica “Diplomat”)	UV-B (310 nm)/1.5 and 7.2 kJ m^–2^/20 cm	4, 7 and 14 days at 4 °C with 90–95% RH	(8)
	total, aliphatic, and indolyl glucosinolates	H2 decreased total GLs content after 2 h, then a subsequent increase; the highest levels of GLs were achieved after 18 h of the UV-B treatment; aliphatic glucoraphanin showed the highest induction in response to UV-B exposure	broccoli (*Brassica oleracea* var. italica, cv. Legacy)	UV-B (290–340 nm)L2/L12: 3.2 W m^–2^, 2.0/12.0 kJ m^–2^ and H2/H12: 5 W m^–2^, 2.0/12.0 kJ m^–2^/30 and 15 cm	2, 6 and 18 h in the dark at 20 °C	(25)
	G1, G2, G3, G4 and G5	both doses of UV-C treated broccoli remained G1 modestly higher than untreated, and significant differences observed at the beginning of the storage; total G2 increased significantly after both UV-C treated, and the titers remained nearly steady throughout the storage; G4 in broccoli treated with both UV-C were higher by the end of the storage after 14 days compared to the control	broccoli (*Brassica oleracea* var. italica, cv. Legacy)	UV-C/0, 1.2, 3.0 kJ m^–2^ (20.4 W m^–2^)/30 cm	4, 7 and 14 days at 4 °C with 90–95% RH	(85)
	glucoiberin, progoitrin, glucoerucin, G1, G2, G3, G4, and G5	UVB reported an increase of 18% after the first UV application, reaching an increase of 44% on the seventh day; UV-C and UV-C+UV-B treatments did not positively affect to the GLs content, and the UV-C+UV-B treatment reported similar amounts to control	broccoli (*Brassica olearacea* var. italica)	UV-B, UV-C, UV-B + UV-C/9 kJ m^–2^ UV-C (26.06 ± 0.37 W m^–2^); 15 kJ m^–2^ UV-B (7.99 ± 0.40 W m^–2^); 9 kJ m^–2^ UV-C + 15 kJ m^–2^ UV-B/17.5 cm	4, 7 and 10 days at 4 °C	(86)
	G1 and G2	G1 was initially increased by 14–25% after UV-B5,, UV-B10, and UV-B15 + UV-C treatments while the remaining UV treatments induced lower or none G1 enhancements; G2 showed higher enhancements than G1 after UV treatments, ranging from 20 to 85%, UV-B10+C combination was the only treatment able to increase the G2 content of Bimi leaves up to 34%; all UV induced higher G1/G2 contents after storage	Bimi broccoli	UV-B, UV-B+UV-C/5, 10, and 15 kJ m^–2^ UV-B (9.27 W cm^–2^); UV-B + 9 kJ m^–2^ UV-C (25.21 W cm^–2^)/17.5 cm	24, 48 and 72 h at 15 °C with 90–95% RH	(53)
	G2 and G5	a distinct decrease in the contents of the G2 and G5 was detected in the UV-B treated samples, approximately 0.3–0.1 mg g^–1^ dm for G2 and 0.27–0.05 mg g^–1^ dm for G5	white cabbage var. Lennox	UV-B (290–315 nm)/0.3–0.4 W m^–2^, 12 h per day/–	2, 4 and 7 d at 4 °C	(75)
betalanins	betanin (Bn) and vulgaxanthin I (Vx)	Bn:Vx ratio increased 55% and 47% in “Monty Rz” and “Belushi Rz, respectively, while no changes on Bn content on both cultivars, Vx content decreased significantly after both 3 and 7 days of storage, regardless of red beet were untreated or subjected to UV-B radiation	red beet (*Beta vulgaris* L. spp. vulgaris “Monty Rz” and “Belushi Rz”)	UV-B/1.23 kJ m^–2^ (17.5 W m^–2^)/17.5 cm	3 and 7 days in the dark at 15 °C with 98–100% RH	(87)

TCC showed a quite different picture, partly depending on the species or UV wavelength. Carrots irradiated with a low dose of UV-C (0.8 kJ m^–2^) initially showed a sharp decrease in TCC and then an increase in TCC during storage, just like the control samples.^46^ A decrease after irradiation could be due to the antioxidant function of carotenoids,^29^ as they were oxidized and no longer detectable. A delayed increase may be due to a positive feedback mechanism. In bell peppers treated with UV-C, TCC increased during storage (15–18 days), mainly due to ripening processes.^17,18^ In the first study, UV-C samples (6.6 kJ m^–2^) showed increased levels the sixth day compared with the control. However, in the second study, the UV-C treated samples (7.0 kJ m^–2^) showed a smaller increase than the control during the 18 day storage period. High irradiation doses may cause excessive oxidative stress, reducing antioxidants instead of leading to a positive feedback mechanism. In red cabbage, the control initially increases, and after four days of storage, the UV-C-treated samples (1.0, 3.0, and 5.0 kJ m^–2^) show an increase in TCC.^56^ Again, a dose dependence was present as the mean irradiation dose (3.0 kJ m^–2^) provided the highest values, but all values were well below the control. The authors argue that UV-C can cause photobleaching and degradation of carotenoids, decreasing the total concentration. In wild rocket, the highest TCC value was recorded with 0.2 kJ m^–2^ UV-B treatment for 45 s under scattered light film.^57^ UV-B irradiation (1.4 kJ m^–2^) of carrot slices of different size showed no variation of TCC; however, the UV-B samples tend to be reduced at day three.^55^ It is possible that the TCC value could be increased by a positive feedback mechanism. Longer doses of UV-A radiation could increase TCC in tomato.^20^ A general statement on the influence of a longer wavelength is not yet possible, as further research is needed. However, results similar to those of UV-C can be expected if comparable doses are used.

### Vitamins

UV radiation affects various phytochemicals. However, very few studies have examined the vitamin content in vegetables after UV irradiation (Table 3). Ascorbic acid (AA), which serves as vitamin C in humans, is present in all plant cells and is the strongest contributor to the cellular redox state.^58^ Therefore, it is an important quencher of ROS because its concentration exceeds that of other antioxidants. Other vitamins such as β-carotene, provitamin A, or tocopherols, vitamin E derivatives, are also potent antioxidants, both of which are fat-soluble. Carotenes protect plant cells by quenching triplet chlorophylls and ROS under excessive light energy conditions.^28^ Tocopherols, on the other hand, are important for stabilizing membranes in plants.^59^

In a study on leek, spinach, and cabbage, the reduction of AA was measured after single (2.46 kJ m^–2^) and multiple (5 × 2.46 kJ m^–2^ = 12.3 kJ m^–2^) UV-C irradiations. The reduction in AA during storage was less pronounced with multiple irradiations than with single or control irradiation.^60^ In studies on cucumber (3.9, 7.7, 11.6 kJ m^–2^), water bamboo (4.2 kJ m^–2^), and bell pepper (6.6 kJ m^–2^) a correlation of AA and UV-C irradiation during storage was not present.^13,17^ The author of the cucumber study explained the lack of an effect by drastic weight loss. The water loss caused by respiration leads to degradation of AA, making any possible influence difficult to detect. In a study of UV-C (10.0 kJ m^–2^) treated bell peppers AA, dehydroascorbic acid (DHA), and 2,2-diphenyl-1-picrylhydrazyl (DPPH) radicals increased only on day 21 of storage. AA and DHA were not measured on day 14 of storage, but DPPH radical inhibition was already elevated.^61^ The control samples showed increased wounding and AA is highly susceptible to oxidation. Nevertheless, the UV-C samples showed higher values, which can be attributed to better shape stability due to the UV-C treatment (see the Texture section) and the physiological response of the fruit.^61^ A beneficial effect of UV radiation was also found in tomato in a review (2.0–8.0 kJ m^–2^).^14^ Membrane damage during storage can lead to increased oxidation of AA.^14,62^

UV-B irradiated parsley (42.0 kJ m^–2^) showed no difference from the control during 6 days of storage.^63^ In ripe green tomatoes, UV-B irradiation had a negative effect after 37 days of storage, as the irradiated samples had 18% lower AA levels at the end of storage (10.0–80.0 kJ m^–2^).^26^ In another study, only the tomato cultivar Money Maker was one of two varieties to show a positive trend for AA in flesh and skin.^64^ Tomatoes received daily UV-B irradiation (6.1 kJ m^–2^) until they were fully ripe (red ripening stage). They were harvested at either the ripe green stage or the envelope stage and thus received a daily UV-B dose between 10 and 22 days. Money Maker is a common commercial cultivar, while the second variety is a mutant with high pigment content (hp-1) and produces higher amounts of lycopene. The hp-1 mutant showed no changes in skin or flesh, except for a lower AA concentration in the flesh of ripe green fruits. Nevertheless, a clear trend is not detectable for any UV species. It could be that lower values can also trigger a positive feedback mechanism, while high values cause too much cell damage, as explained by Andrade Cuvi et al.^61^ However, further research is needed here, especially for UV-B and UV-A.

In a study of amaranth leaves, vitamin E was measured in the form of α-tocopherol. During storage, α-tocopherol was hardly affected by UV-C irradiation, except after 14 days at 5 °C where leaves treated with UV-C (1.7 and 3.4 kJ m^–2^) had higher vitamin E content than the control group.^49^ Storage conditions (20 or 5 °C) had a greater effect than UV-C irradiation, as storage temperature (5 °C) helped to maintain α-tocopherol content longer.^49^ Plant physiological activities are reduced by low temperatures and thus also inhibit tocopherol synthesis, while high temperatures (20 °C) promote synthesis.^49^ The authors claim that this is an explanation not only for the difference in concentration at the beginning of storage but also for the late UV-C effect. Since UV-C radiation causes ROS production in leaves, this triggers tocopherol production only after some time. Further studies are needed, especially on the influence of UV radiation on the vitamin E content of vegetables. This study gives only a first impression. Again, further research is needed to investigate UV-A and UV-B radiation after harvest on vegetables.

### Phenolic Compounds

Phenolic compounds are the largest group of secondary plant metabolites and are subdivided into several subgroups, such as phenolic acids, flavonoids, and tannins.^65^ Their function in plants ranges from defense, especially against high radiation, to coloration. Therefore, phenols contribute to the overall fitness of plants.^66^ They are also desirable as antioxidants in food. Thus, their induction by UV treatment would be a great benefit to the nutritional value of vegetables.

UV radiation has an enhancing effect on the phenolic compounds; especially UV-C leads to the accumulation of various phenolics (Table 3).^19,50,52,67−70^ A review by Urban et al.^71^ summarized that UV-C light induces phenylalanine ammonia lyase activity at the post-transcriptional level, along with various enzymes of the phenolic biosynthetic pathway, depending on the species and variety. Studies on carrots showed a simple increase in 6-methoxymellein (0.9 kJ m^–2^).^67,68^ In contrast, studies analyzing several different phenolic compounds showed that accumulation is more complex. Wei et al.^19^ found an increase in some phenolic compounds (naringenin, luteolin, and apigenin) with prolonged UV-C irradiation (0.9–3.6 kJ m^–2^) of pigeon pea leaves, whereas others initially increased and then decreased (orientin, apigenin-6,8-di-C-α-l-arabinopyranoside, pinostrobin, longistyline C, and cajaninstilbenic acid). One phenolic compound, pinostrobin chalcone, was not affected by UV-C irradiation. Similar results were published for a storage experiment with UV-C irradiated tomatoes (4.0 kJ m^–2^): Several phenolic compounds increase during storage (*p*-coumaric acid, caffeic acid, ferulic acid, chlorogenic acid, and protocatechuic acid), while some decrease (naringenin and quercetin).^51^ In the same experiment, gallic acid and rutin initially increased during storage and then had levels similar to control samples after 35 days. Irradiation with UV-C (1.0, 3.0, and 5.0 kJ m^–2^) can also lead to the formation of new phenolic compounds: e.g., in a study on red cabbage control samples accumulated 11 anthocyanin compounds, while samples treated with UV-C had 15 compounds.^50^ Control and UV-C samples had the same major anthocyanin compounds, but the UV-C samples had four additional minor components (cyanidin (Cy)-3-*O*-glucoside-5-*O*-glucoside, Cy-3-*O*-(*p*-coumaroyl)-diglucoside-5-*O*-glucoside, Cy-3-*O*-(feruloyl)-glucoside-5-*O*-glucoside, and Cy-3-*O*-(sinapoyl)-glucoside-5-*O*-glucoside). This study also showed that some anthocyanins increased with increasing UV-C dose (Cy-3-*O*-glucoside-5-*O*-glucoside, Cy-3-*O*-(feruloyl)-glucoside-5-*O*-glucoside, and Cy-3-*O*-(sinapoyl)-glucoside-5-*O*-glucoside), while others decreased during storage (Cy-3-*O*-diglucoside-5-*O*-glucoside, Cy-3-*O*-(feruloyl)-triglucoside-5-*O*-glucoside, Cy-3-*O*-(sinapoyl)-triglucoside-5-*O*-glucoside, and Cy-3-*O*-(sinapoyl)-diglucoside-5-*O*-glucoside). The highest sum of anthocyanins among the three treatments was accumulated at 3.0 kJ m^–2^ on the eighth day of 12 days of storage.^50^ In another study, the storage condition also played a role, as flavonoids increased at 5 °C for all doses (1.7 and 3.4 kJ m^–2^) on the second day. At the highest dose at 20 °C, there was no increase until day four.^49^ In the same study, all phenolic acids were increased regardless of the dose and temperature. The authors expected a higher increase at 20 °C because higher temperatures increase enzymatic activities and, thus, accelerate synthesis. However, they argue that responses to UV-C, especially flavonoid accumulation, depend on the physiological stage of development as well as the species and cultivar. An increasing effect of UV radiation on phenolic compound concentrations is already more or less common sense, as described in reviews.^72,73^ Nevertheless, the question remains why only some phenolic compounds are increased by UV irradiation and not all. Because of the great diversity of phenolic compounds, they have different roles in the plant, such as defense mechanisms against pathogens, parasites, and predators, reproduction and growth, and contribution to plant color.^66,74^ Consequently, only those phenolic compounds that help to remedy the destructive effects of UV radiation are increased, especially those with high antioxidant activity to quench ROS.

Irradiation with UV-B or UV-A showed trends similar to that of irradiation with UV-C. In one of the studies mentioned above, the effect of UV-B and UV-A on pigeon pea leaves was also investigated.^19^ Some phenolic compounds increase more with UV-B irradiation (pinostrobin chalcone, pinostrobin, longistyline C, and cajaninstilbene acid). Some of the phenolics that increased with increasing UV-C dose were not affected by UV-B (0.2–0.9 kJ m^–2^) (naringenin, luteolin, and apigenin).^19^ Irradiation with UV-A (0.05–0.22 kJ m^–2^) showed similar trends to UV-B, but less marked.^19^ In spinach, UV-B and UV-A have different effects. After three days of irradiation with UV-B (42.0 kJ m^–2^), the levels of *p*-coumaric acid increased, and jaceidin was newly formed. The same treatment with UV-A (165 kJ m^–2^) showed no difference from the control samples.^63^ UV-A and UV-B light has an enhancing effect on flavonoids after three days of irradiation: the content of kaempferol glycosides increased in radish shoots. In parsley, the aglycone apigenin increased 7-fold under UV-B and 2.5-fold under UV-A compared to the control. Vitexin content in Indian spinach increased with UV-B irradiation on the third day and with UV-A irradiation on the sixth day.^63^ This study clearly shows that flavonoids increase after UV treatment and thus play a protective role against UV-B and UV-A radiation. However, flavonoids increase differently in different species due to individual adaptation to abiotic stress. Also, in a study on broccoli, only 1-sinapoyl-2-feruloyl gentiobiose was increased, whereas other hydroxycinnamic acids remained unchanged (1,2-disinapoyl-2-feruloyl gentiobiose, 1,2,2-trisinapoyl gentiobiose, 1,2-diferuloyl gentiobiose, and 1,2-disinapoyl gentiobiose) regardless of the applied UV-B doses (1.5 and 7.2 kJ m^–2^).^8^ In a study on carrots, the effect of a different pruning style before UV-B irradiation (1.4 kJ m^–2^) was analyzed. An increase in chlorogenic acid was observed in all carrots after three days of storage following UV-B irradiation.^55^ The different cut types had an effect, as carrot chips responded the most and baby carrots the least. This may be due to the large surface area to volume ratio and greater wounding of the carrot chips. This suggests that pretreatment affects the sensitivity to UV radiation. UV-B irradiation was also able to stimulate the synthesis of newly formed phenols in white cabbage, a plant low in hydroxycinnamic acids (mainly sinapic acid derivatives) and lacking flavonoids.^75^ After 2 or 4 days of UV-B irradiation (13.0–17.0 kJ m^–2^), newly formed hydroxycinnamic acid glycosides such as coumaroyl glycoside, feruloyl glycoside, and caffeoyl glycoside were found. *De novo* formation of the flavonoid quercetin triglycoside was also higher in the outer leaves than in the newly formed hydroxycinnamoyl glycosides. The authors suggest that this is due to the activation of various enzymes in the biosynthetic pathway of polyphenols under UV-B treatment after harvest. Among others, phenylalanine ammonia lyase and cinnamate 4-hydroxylase are activated for the formation of early phenols and, in addition, hydroxylation and methylation reactions are promoted.^75^ The reduced and altered synthesis of phenolic compounds by UV-A and UV-B radiation is expected, as these wavelengths are less energetic and therefore cause less cell damage and ROS production. Therefore, the need to synthesize protective phenolic compounds is less. UV-A and UV-B are part of natural radiation, but UV-C is blocked by the ozone layer. Therefore, plants have special adaptation mechanisms to these UV wavelengths. Nevertheless, UV-C and UV-B and UV-A seem to be effective in increasing the content of phenolic compounds in the postharvest.

### Carotenoids

Carotenoids are pigments that range in color from yellow to red. The color is the result of the physical property of a polyene chain with multiple conjugated double bonds that act as a chromophore.^29^ Some carotenoids, such as β-carotene, lutein, and zeaxanthin, are involved in light collection at the photosynthetic membranes of the chloroplast. In the presence of excessive light, they can protect the photosynthetic apparatus by quenching triplet chlorophylls, singlet oxygen, and ROS.^28,29^ Induction of carotenoids in vegetables is beneficial for human health.

There have not been many studies analyzing individual carotenoids after UV irradiation in vegetables (Table 3). The few studies that investigated the effect of individual carotenoids mostly used tomato. They were already reviewed with no clear trend. In the review,^14^ several studies found a reduction in lycopene and β-carotene with UV-C radiation (3.7–4.2 kJ m^–2^), while other studies found no effect (4.2–6.1 kJ m^–2^). Two of the eleven studies in this review found an increasing effect, but mainly for lycopene (1.0–13.7 kJ m^–2^)^14^ One of these studies found a positive effect for lycopene, β-carotene, and lutein concentration in the skin of a tomato variety, in addition to an increase for lycopene in the flesh. In contrast, no effect was observed in a particularly lycopene-rich mutant (Hp-1). The two cultivars studied received 6.1 kJ m^–2^ UV-B daily after being harvested immature until they were fully ripe.^64^ This study showed that the cultivar choices may be one of the main reasons why conflicting results sometimes occur. In a more recent study, increases in lycopene, β-carotene, and lutein were found in several cases at different UV-A wavelengths. However, this was significant only when a long irradiation time such as 180 min (2.0 kJ m^–2^) or 360 min (4.0 kJ m^–2^) was used.^20^ There were two studies by Gogo et al. on amaranth and African nightshade.^39,49^ UV-C irradiation initially decreased the content of carotenoids, but as the storage time progressed, the content increased in amaranth and African nightshade. Higher irradiation (3.4 kJ m^–2^) resulted in higher β-carotene and lutein content on days 2 and 4, whereas lycopene content increased on day 4 and decreased thereafter.^49^ These contradictory effects may be due to a variety of reasons. Several studies have shown that different tomato cultivars respond differently to abiotic stress.^76−78^ In addition, some of the carotenoid synthesis steps are light-mediated, such as carotene isomerase or lycopene-β-cyclase.^14^ The latter converts lycopene to β-carotene, which only reduces the lycopene content. This explains why some studies found an increasing effect but most found a decreasing effect. The ability to suppress excessive light and ROS as an antioxidant is most likely responsible for their degradation. There is little evidence on the effects of UV-B and UV-A radiation on postharvest tomatoes, except for the studies cited. Therefore, further research is needed for tomatoes and especially for UV-B and UV-A.

### Chlorophyll

The chlorophyll molecules are the most abundant molecules and are essential for photosynthesis, which allows plants to absorb energy from light. In photosynthetic organisms, chlorophylls a and b predominate. Decolorization would mean a significant loss of quality in vegetables.

UV-C radiation has the potential to inhibit chlorophyll loss in leaves and inflorescences. In spinach, broccoli, leeks, and cabbage, degradation is reduced by UV-C irradiation (Table 3).^12,24,41,60^ Higher irradiance levels are better at preventing chlorophyll degradation (4.0–14.0 kJ m^–2^).^12,41^ However, the highest dose (14.0 kJ m^–2^) delayed chlorophyll degradation while increasing the pheophytin content. This resulted in a surface color similar to that of the control samples after a few days of storage.^12^ In this study, decreased chlorophyllase and MDS activity were observed but not chlorophyll peroxidase activity. The authors relate the decreased enzyme activity to UV-C irradiation, as has been shown in other studies.^12^ Also in a study by Liao et al.^60^ multiple irradiations (5 × 2.46 kJ m^–2^ = 12.3 kJ m^–2^) showed the best results in preventing chlorophyll loss in leeks, spinach, and cabbage, while single irradiation (2.46 kJ m^–2^) did not prevent degradation or did so to a lesser extent. Prolonged storage (10 days or longer) increased chlorophyll content in amaranth and African nightshade, while shorter storage times at 20 °C showed no effect in African nightshade and there was an increase in chlorophyll a and b in amaranth (1.7 and 3.4 kJ m^–2^).^39^ Contrasting results were seen in a study of cucumbers (4.0–14.0 kJ m^–2^), in which irradiated samples had lower chlorophyll content from the third day of storage to the end of the experiment (tenth day).^13^ However, on the day of irradiation, the values of the irradiated cucumber fruit samples were higher than those of the control. The authors suggested that a high respiration rate due to high turnover may be responsible for chlorophyll degradation.^13^ In conclusion, UV-C radiation has a great potential to inhibit chlorophyll degeneration. However, it may also inhibit chlorophyll biosynthesis in water bamboo. In this vegetable, the green outer layer is peeled, and it should remain white. The content of chlorophylls a and b was higher in the control samples than in the UV-C treated shoots. The author claims that this shows that UV-C treatment (4.2 kJ m^–2^) can inhibit chlorophyll biosynthesis in water bamboo.^15^

There are few studies that have investigated the effect of UV-B and UV-A irradiation on chlorophyll in vegetables after harvest. There is one study with UV-A irradiation on three red ripe tomato cultivars.^20^ Longer irradiation periods with different UV-A wavelengths led to an increase in total chlorophyll in all cultivars. This suggests that UV-A may also have a protective effect against chlorophyll degeneration, although only trace amounts of chlorophyll are detectable in red ripe tomatoes.^20^ In tomatoes, plastids change from green chloroplasts to red chromoplasts during the ripening process.^79^ This process was slowed by UV irradiation. Also, in two studies by Aiamla-or et al.^7,80^ broccoli showed delayed yellowing of the flower after UV-B irradiation (8.8 to 19.0 kJ m^–2^). However, slight differences were observed. The flowers of the cultivar Pixel yellowed faster than the flowers of “Sawayutaka”. This not only suggests that UV-B may have the same effect as UV-C but also shows that the choice of cultivar is important for the outcome. In a study of wild arugula, plants were grown under three different cover films with different UV-B transmittances, and fresh-cut arugula was irradiated with UV-B for 45 s after harvest.^57^ For all three different films, UV-B treatment (0.2, 0.7, 1.5, and 3.0 kJ m^–2^) for 45 s resulted in higher chlorophyll a levels and was different from the other treatments of each film, also indicating a degradation of chlorophylls when a longer UV-B treatment was applied.^57^ However, parsley showed no change in chlorophyll content compared with the control after UV-B and UV-A irradiation (42.0 kJ m^–2^).^63^ Other phytochemicals measured, such as moisture and AA, were also not changed. This suggests that parsley may not be very susceptible to UV radiation. UV-B and UV-A need to be studied further to understand their effects on chlorophyll.

### Glucosinolates

Glucosinolates (GLs) are also an important class of bioactive plant compounds. They are derived from amino acids and belong to a large group of secondary metabolites containing N and S. They have a common structure that includes a β-thioglucose group, a sulfonated oxime unit, and a variable aglycone side chain derived from the various amino acids.^81^ Based on the amino acid precursors, GLs are mainly classified into three groups: aliphatic, aromatic, and indolic glucosinolates.^82^ GLs are mainly abundant in the Brassicaceae family, including *Brassica oleracea* (i.e., broccoli, cabbage, cauliflower, kale, Brussels sprouts), *Brassica rapa* (i.e., turnip, Chinese cabbage, pak choi), *Brassica napus* (i.e., oil seedrape), *Ranphanus sativus* (radish), and *Sinapis alba* (mustard).^83^

The biosynthesis of glucosinolates can be activated by biotic and abiotic stress.^84^ Duarte-Sierra et al.^85^ reported that the glucobrassicin content of broccoli florets increased by both 1.2 and 3.0 kJ m^–2^ UV-C treatments, and the concentrations remained nearly steady throughout the storage period compared to the control. At the same time, the concentrations of 4-hydroxyglucobrassicin (4-OH-GLB) in broccoli treated with 1.2 and 3.0 kJ m^–2^ UV-C radiation were higher by the end of the storage after 14 days compared to the control.^85^ UV-B and UV-C light doses and storage times differentially tailor GLs profiles in broccoli florets, and UV-B could enhance the content of GLs after storage (Table 3).^8,25,85,86^ It was reported that hormetic and higher doses of UV-B (1.5 and 7.2 kJ m^–2^) increased the total glucobrassicins by 18%, and 22%, respectively, relative to the control.^8^ Aliphatic glucoraphanin showed the highest induction in response to UV-B exposure.^25^ Besides, storage time has a positive effect on GL accumulation in broccoli florets. For instance, high-intensity (5.0 W m^–2^) and low-dose (2.0 kJ m^–2^) UV-B radiation in postharvest could decrease total GL content after 2 h, though a subsequent increase was observed and the highest levels of GLs were achieved after 18 h of UV-B treatment.^25^ In Bimi broccoli, single UV-B and UV-B combined with UV-C radiation were applied and it was reported that all UV treatments induced higher glucoraphanin and glucobrassicin contents after storage compared to nonirradiated samples in such broccoli florets; among them 5.0 kJ m^–2^ of UV-B radiation induced the highest glucoraphanin/glucobrassicin in florets after 72 h regarding their respective initial levels.^53^ On the contrary, another study showed that decreasing contents were observed for the glucosinolates glucobrassicin and 4-methoxyglucobrassicin in the UV-B treated (13.0–17.0 kJ m^–2^) white cabbage but there was formation of the degradation products dihydroascorbigen and dihydro-4-methoxyascorbigen, which might be related to cell shrinking as mechanical damage.^75^ It can be concluded that UV radiation can increase glucosinolates.

### Betalains

Betalains are an excellent substitute for thermolabile anthocyanins as food colorants. They contain two groups of water-soluble pigments: red-purple betacyanidins and yellow betaxanthins. Betacyanidins are conjugates of cyclo-dihydroxyphenylalanine and betalamic acid, and betaxanthins are conjugates of amines or amino acids and betalamic acid.^87^

Betalains are also an important group of plant secondary metabolites whose accumulation can be triggered by abiotic factors or environmental stressors. To our knowledge, water and salt stress can induce the pigments, but there have been few studies on betalanines under UV stress on vegetables, none of which were conducted with UV-C (Table 3).^88,89^ In the postharvest environment, a low UV-B treatment (1.23 kJ m^–2^) of the beetroots, followed by short-term storage for three and seven days, increased the ratio of betalain to vulgaxanthin in the beets without adversely affecting beet quality, whereas neither three and seven day storage nor UV-B irradiation altered the betalain content of ”Monty Rz” and ”Belushi Rz” beets.^87^ However, after three and seven days of storage, vulgaxanthin I content in beets decreased, regardless of whether the samples were control or UV-B treated.^87^ Betalain pigments may also play some role in protecting against UV stress, but further evidence is needed to support this hypothesis.

### Enzymes

Enzymes act as biocatalysts and, thus, accelerate chemical reactions in living organisms. Like any other catalyst, enzymes do not alter the equilibrium and are not changed by chemical reaction. However, enzyme activity is altered by other molecules that act as either activators or inhibitors. UV radiation has the potential to inactivate enzymes by denaturing them and consequently reducing their activity.^90^

UV-C treatment increased the activities of red cabbage antioxidant enzymes, especially after eight days of storage. Only at the beginning were superoxide dismutase and peroxidase increased at lower UV-C irradiances (1.0, 3.0, and 5.0 kJ m^–2^).^56^ In the same study, H_2_O_2_ was also measured; on the first day of storage, levels were increased at all UV-C irradiance doses compared with the control. After four days of storage, only the lowest dose showed increased H_2_O_2_ levels, whereas no differences were detectable on the eighth day. This suggests that the increased activity of antioxidant enzymes reduces the levels of ROS, such as H_2_O_2_. In UV-C treated bell peppers, levels of catalase and ascorbate peroxidase increased immediately and after seven days of storage, while superoxide dismutase and guaiacol peroxidase increased only after seven days of storage.^61^ In another study on bell pepper, some enzymes (catalase and superoxide dismutase) increased shortly after UV-C irradiation and remained until the twelfth or fifteenth day, while guaiacol peroxidase was increased only on the third day and ascorbic acid peroxidase was not increased at all.^17^ Similarly, in water bamboo, only the enzyme catalase was increased after UV-C treatment during the eight days of the study, but significantly only from the fourth to the sixth day. Guaiacol peroxidase and ascorbate-dependent peroxidase were not affected by UV-C treatment (4.24 kJ m^–2^).^15^ Again, it appears that the different species and cultivars do not respond uniformly to UV-C but have individually elevated activities of antioxidant enzymes for the detoxification of superoxide and hydrogen peroxide. Studies of UV-A and UV-B are missing in this context so far.

Phenolic biosynthesis enzymes are positively affected by UV radiation. In fresh-cut cabbage, the expression of most anthocyanin biosynthetic and regulatory genes was slightly upregulated by different UV-C doses in UV-C treated samples (1.0, 3.0, and 5.0 kJ m^–2^).^50^ Most of the genes showed their maximal expression levels at day 12. There was a dose dependence, as most anthocyanin biosynthetic genes were most increased at a UV-C dose of 3.0 kJ m^–2^. The gene responsible for the increased expression of anthocyanin biosynthesis was PAP1, which was immediately and dramatically upregulated by UV-C treatment in cabbage. This gene, PAP1, was reported to activate anthocyanin biosynthesis in *Arabidopsis thaliana* by regulating the expression of phenylalanine ammonia lyase and chalcone synthase, among others. The authors argue that it may also be responsible for the increase in cabbage.^50^ In tomato, phenylalanine ammonia lyase was upregulated by UV-C irradiation (4.0 kJ m^–2^), among others such as cinnamate 4-hydroxylase or 4-coumarate CoA ligase.^51^ Phenylalanine ammonia lyase was also upregulated in UV-C treated water bamboo compared with the control, but only on the second and eighth day (4.2 kJ m^–2^).^15^ Again, most enzymes showed the highest concentration between the seventh and 28th days of storage. This positive influence of UV radiation on the enzymes of phenolic biosynthesis was also evident in the results of the phenolic compounds. Not all phenolics increased after UV irradiation, and some increased later than others. This shows the great variability with which plants respond to abiotic stress. One possible reason for a delayed increase is the denaturation of some enzymes by UV radiation.^90^ However, this has been demonstrated for juices, so it may be less important for intact vegetables. Irradiation with UV-B (42.0 kJ m^–2^) also enhanced the phenolic biosynthesis in parsley. Phenylalanine ammonia lyase expression increased after 6 h and was maintained for 24 h. In addition, the enzymes cinnamic acid 4-hydroxylase, 4-coumarate CoA ligase, chalcone synthase, and flavone synthase were increased within the 72 h study period. As a result, cinnamic acid production increased in parsley leaves.^63^ However, in two other studies, phenolic biosynthesis enzymes were not affected.^8,32^ In one of the two studies, lignification of edible bamboo shoots was delayed, possibly due to decreased activities of the enzymes phenylalanine ammonia lyase, 4-coumarate CoA ligase, cinnamyl alcohol dehydrogenase, and peroxidase.^32^ Phenolic compounds are precursors of lignin, and an increased lignin content in edible bamboo is undesirable. The second study, which analyzed the activity of phenolic biosynthetic enzymes in broccoli, was also unaffected by UV-B (1.5 and 7.2 kJ m^–2^). They showed a low relative gene expression in the treated florets. Most hydroxycinnamic acids (1,2-disinapoyl-2-feruloyl gentiobiose, 1,2,2-trisinapoyl gentiobiose, 1,2-diferuloyl gentiobiose, and 1,2-disinapoyl gentiobiose) were also not affected. Only the concentration of 1-sinapoyl-2-feruloyl gentiobiose increased, but for both doses.^8^ However, enzyme activity was measured 6 h after irradiation, but the concentration of hydroxycinnamic acids was measured four, seven, and 14 days later. Thus, the different results can be explained by different measurement times. These two studies do not fit into the general picture of a positive influence of UV radiation on phenolic compounds (see Phenolic Compounds section). It is possible that the results of these two studies would have been different at other time points. Nevertheless, this review has shown that there are varieties and species that are less sensitive to UV radiation.

Chlorophyll-degrading enzymes in UV-C treated broccoli were reduced during six days of storage, although at different times (10.0 kJ m^–2^).^12^ Chlorophyllase increased on the second day in the control samples but not in the UV-C treated samples. Peroxidase increased in both UV-C and control samples but increased more in the control on the sixth day. Mg-dechelatase increased only in the UV-C treated samples but decreased below the control value from the fourth day. Therefore, in this study by Costa et al.^12^ the decreased chlorophyll degradation can be attributed to the decreased activities of chlorophyll-degrading enzymes. The authors argue that these enzymes are stimulated by ethylene and that ethylene is reduced by UV irradiation. This also allows a higher chlorophyll content to be maintained during storage. Also, in a study by Büchert et al.^24^ UV-C treated broccoli (10.0 kJ m^–2^) showed higher chlorophyll content compared to the control after five days of examination. While the first chlorophyllase (BoCLH1) showed decreased expression, the second (BoCLH2) showed twice the expression compared with the control on the fifth day of the study. At the same time, the expression of pheophytinase (BoPPH) was reduced by almost half in the UV-C treated samples. This result again shows the variation of enzymes, although the same result was obtained. Irradiation of broccoli with UV-B also showed different results for chlorophyll-degrading enzymes. First, the expression of chlorophyllase genes (BoCLH1, BoCLH2, and BoCLH3) increased immediately after UV-B treatment (19.0 kJ m^–2^). On the fourth day, the expression of BoCLH1 was reduced in the UV-B treated samples, whereas the expression of the other two chlorophyllase genes was not changed compared with that of the control. Pheophytinase (BoPPH) showed a small reduction in UV-B treatment on the second and fourth day. The treated broccoli showed a two day delay in senescence.^80^ These three studies confirm that UV-C and UV-B can delay chlorophyll degradation in broccoli. The studies also show that there is variability in the expression of chlorophyllase genes. Büchert et al.^24^ concluded that BoPPH has the best potential to predict chlorophyll degradation. The expression of this enzyme is reduced when the chlorophyll content of treated broccoli has higher values than that of the control.

An accumulation of indole-type glucosinolates was observed in broccoli after hormetic and higher UV-B exposure (1.5 and 7.2 kJ m^–2^), while a lower accumulation of aliphatic-type glucosinolates was observed.^8^ In this study, some precursor amino acids and the expression of genes related to the biosynthetic pathway of glucosinolates were also investigated. Postharvest exposure to UV-B radiation showed that several genes of the glucosinolate synthesis pathway were overexpressed.^8^ Among them, the most relevant was the overexpression of tryptophan N-hydroxylase (CYP79B3), which was increased 6-fold in flowers exposed to the hormetic UV-B dose (1.5 kJ m^–2^) and 10-fold in the higher UV-B dose (7.2 kJ m^–2^). At the same time, the higher UV-B dose resulted in a 3-fold overexpression of dihomethionine N-hydroxylase (CYP79F1). This result suggests that the target of UV-B is probably the branching pathway of indole glucosinolates.^8^ In another paper by the same author, three genes associated with the glucosinolate pathway after UV-C irradiation on postharvest broccoli florets were studied. The relative gene expression of enzymes encoding glucosinolate biosynthesis in flowers was affected by UV-C.^85^ In this study, the results showed that the genes encoding phenylalanine N-hydroxylase (CYP79A2) and tryptophan N-hydroxylase (CYP79B3) were increased 2.6- and 3.7-fold, respectively, by the high UV-C dose of 3.0 kJ m^–2^ compared with the control, whereas no significant overexpression of dihomo-methionine N-hydroxylase (CYP79F1) was observed.^85^ However, overexpression of CYP79B3 by 3.1-fold was also observed in flowers exposed to the hormetic UV-C dose (1.2 kJ m^–2^). A similar trend was observed at days two and four after UV-C exposure, where significant overexpression of CYP79B3 was measured. The changes in CYP79B3 expression were associated with the abundance of glucosinolates.^85^ These results suggest that the use of a high dose of UV-C as a postharvest treatment may not only preserve broccoli floret quality but also enhance phytonutrients during storage.

These results suggest that the use of a hormetic dose of UV-C as a postharvest treatment may not only preserve broccoli floret quality but also enhance phytonutrients during storage.

## Conclusion

Ultraviolet irradiation in postharvest as a nonchemical, innovative light treatment is a promising illumination to extend the shelf life and improve the nutritional quality of vegetables. In general, there are a large number of results using UV-C. However, other wavelengths such as UV-B, UV-A, and even violet and blue light are rarely studied and used. One reason for this could be lower efficiency in reducing the microbial load. However, UV-A and UV-B wavelengths are less harmful to humans and plants, so more attention could be paid to their effect on vegetables, especially leafy vegetables. It will become easier to study the singular effects of UV-A, as special luminaires, but especially the development of narrow-band UV LEDs will make it possible to decouple UV-B. The so far small number of UV-A studies will increase significantly in the next few years. In combination with UV-C treatment, these wavelengths could be helpful for the increase in anthocyanins and other antioxidants. From the results, it appears that there is an optimal UV dose. Doses that are too high lead to loss of antioxidants, and doses that are too low have no effect. It is becoming clear that there are effects of species and cultivars, possibly due to morphological characteristics and a certain phytonutrient status before UV treatment. Rather unanswered is the question of whether longer treatments at lower intensities or shorter treatments at higher intensities are more beneficial. Ultimately, postharvest UV treatments can help reduce vegetable loss due to rot and, if tailored to the needs of the vegetable, improve the quality during storage. To date, no clear protocols can be derived and must be tested individually for each vegetable. Therefore, more studies that are systematic are needed.

## References

[ref1] Wang X.; Ouyang Y.; Liu J.; Zhu M.; Zhao G.; Bao W.; Hu F. B. Fruit and Vegetable Consumption and Mortality from All Causes, Cardiovascular Disease, and Cancer: Systematic Review and Dose-Response Meta-Analysis of Prospective Cohort Studies. BMJ 2014, 349 (July), 1–14. 10.1136/bmj.g4490. PMC4115152 25073782

[ref2] Mentella M. C.; Scaldaferri F.; Ricci C.; Gasbarrini A.; Miggiano G. A. D. Cancer and Mediterranean Diet: A Review. Nutrients 2019, 11 (9), 2059 10.3390/nu11092059. 31480794 PMC6770822

[ref3] Pomerleau J.; Lock K.; Knai C.; McKee M. Effectiveness of Interventions and Programmes Promoting Fruit and Vegetable Intake. Eur. J. Public Health 2004, 14.

[ref4] Maharaj R. Effects of Abiotic Stress (UV-C) Induced Activation of Phytochemicals on the Postharvest Quality of Horticultural Crops. Phytochem. - Isol. Characterisation Role Hum. Heal 2015, 10.5772/60050.

[ref5] Zhang W.; Jiang W. UV Treatment Improved the Quality of Postharvest Fruits and Vegetables by Inducing Resistance. Trends Food Sci. Technol. 2019, 92, 71–80. 10.1016/j.tifs.2019.08.012.

[ref6] Civello P. M.; Villarreal N.; Lobato M. E. G.; Martínez G. A. Physiological Effects of Postharvest UV Treatments: Recent Progress. Stewart Postharvest Rev. 2014, 10 (3), 1–6.

[ref7] Aiamla-or S.; Yamauchi N.; Takino S.; Shigyo M. Effect of UV-A and UV-B Irradiation on Broccoli (Brassica Oleracea L. Italica Group) Floret Yellowing during Storage. Postharvest Biol. Technol. 2009, 54 (3), 177–179. 10.1016/j.postharvbio.2009.07.006.

[ref8] Duarte-Sierra A.; Munzoor Hasan S. M.; Angers P.; Arul J. UV-B Radiation Hormesis in Broccoli Florets: Glucosinolates and Hydroxy-Cinnamates Are Enhanced by UV-B in Florets during Storage. Postharvest Biol. Technol. 2020, 168 (April), 111278 10.1016/j.postharvbio.2020.111278.

[ref9] Collazo C.; Noguera V.; Aguiló-Aguayo I.; Abadias M.; Colás-Medà P.; Nicolau I.; Viñas I. Assessing Water-Assisted UV-C Light and Its Combination with Peroxyacetic Acid and Pseudomonas Graminis CPA-7 for the Inactivation and Inhibition of Listeria Monocytogenes and Salmonella Enterica in Fresh-Cut “Iceberg” Lettuce and Baby Spinach Leaves. Int. J. Food Microbiol. 2019, 297, 11–20. 10.1016/j.ijfoodmicro.2019.02.024. 30852362

[ref10] Allende A.; McEvoy J. L.; Luo Y.; Artes F.; Wang C. Y. Effectiveness of Two-Sided UV-C Treatments in Inhibiting Natural Microflora and Extending the Shelf-Life of Minimally Processed “Red Oak Leaf” Lettuce. Food Microbiol. 2006, 23 (3), 241–249. 10.1016/j.fm.2005.04.009. 16943010

[ref11] Allende A.; Artés F. UV-C Radiation as a Novel Technique for Keeping Quality of Fresh Processed “Lollo Rosso” Lettuce. Food Res. Int. 2003, 36 (7), 739–746. 10.1016/S0963-9969(03)00054-1.

[ref12] Costa L.; Vicente A. R.; Civello P. M.; Chaves A. R.; Martínez G. A. UV-C Treatment Delays Postharvest Senescence in Broccoli Florets. Postharvest Biol. Technol. 2006, 39 (2), 204–210. 10.1016/j.postharvbio.2005.10.012.

[ref13] Imaizumi T.; Yamauchi M.; Sekiya M.; Shimonishi Y.; Tanaka F. Responses of Phytonutrients and Tissue Condition in Persimmon and Cucumber to Postharvest UV-C Irradiation. Postharvest Biol. Technol. 2018, 145 (June), 33–40. 10.1016/j.postharvbio.2018.06.003.

[ref14] Mditshwa A.; Magwaza L. S.; Tesfay S. Z.; Mbili N. C. Effect of Ultraviolet Irradiation on Postharvest Quality and Composition of Tomatoes: A Review. Journal of Food Science and Technology 2017, 54, 3025–3035. 10.1007/s13197-017-2802-6. 28974786 PMC5603004

[ref15] Wen B.; Cheng Z.; Hu Y.; Boon-Ek Y.; Wongs-Aree C.; Supapanich S. Ultraviolet-C Treatment Maintains Physicochemical Quality of Water Bamboo (Zizania Latifolia) Shoots during Postharvest Storage. Postharvest Biol. Technol. 2019, 152, 65–72. 10.1016/j.postharvbio.2019.02.017.

[ref16] Alfaro L.; Soler-Segura R.; Jacquin C.; Juan M.; Elorrieta M. A.; Valenzuela J. L. Yellow Bell Pepper Fruit Response to Postharvest Application of Ultraviolet Radiation. In Acta Horticulturae 2018, 1194, 815–821. 10.17660/ActaHortic.2018.1194.115.

[ref17] Promyou S.; Supapvanich S. Effect of Ultraviolet-C (UV-C) Illumination on Postharvest Quality and Bioactive Compounds in Yellow Bell Pepper Fruit (Capsicum Annuum L.) during Storage. AFRICAN J. Agric. RESEEARCH 2012, 7 (28), 4084–4096. 10.5897/ajar12.242.

[ref18] Vicente A. R.; Pineda C.; Lemoine L.; Civello P. M.; Martinez G. A.; Chaves A. R. UV-C Treatments Reduce Decay, Retain Quality and Alleviate Chilling Injury in Pepper. Postharvest Biol. Technol. 2005, 35 (1), 69–78. 10.1016/j.postharvbio.2004.06.001.

[ref19] Wei Z. F.; Luo M.; Zhao C. J.; Li C. Y.; Gu C. B.; Wang W.; Zu Y. G.; Efferth T.; Fu Y. J. UV-Induced Changes of Active Components and Antioxidant Activity in Postharvest Pigeon Pea [Cajanus Cajan (L.) Millsp.] Leaves. J. Agric. Food Chem. 2013, 61 (6), 1165–1171. 10.1021/jf304973f. 23320913

[ref20] Dyshlyuk L.; Babich O.; Prosekov A.; Ivanova S.; Pavsky V.; Chaplygina T. The Effect of Postharvest Ultraviolet Irradiation on the Content of Antioxidant Compounds and the Activity of Antioxidant Enzymes in Tomato. Heliyon 2020, 6 (1), e03288 10.1016/j.heliyon.2020.e03288. 32021939 PMC6992987

[ref21] Birmpa A.; Sfika V.; Vantarakis A. Ultraviolet Light and Ultrasound as Non-Thermal Treatments for the Inactivation of Microorganisms in Fresh Ready-to-Eat Foods. Int. J. Food Microbiol. 2013, 167 (1), 96–102. 10.1016/j.ijfoodmicro.2013.06.005. 23827815

[ref22] Kim Y. H.; Jeong S. G.; Back K. H.; Park K. H.; Chung M. S.; Kang D. H. Effect of Various Conditions on Inactivation of Escherichia Coli O157:H7, Salmonella Typhimurium, and Listeria Monocytogenes in Fresh-Cut Lettuce Using Ultraviolet Radiation. Int. J. Food Microbiol. 2013, 166 (3), 349–355. 10.1016/j.ijfoodmicro.2013.08.010. 24021819

[ref23] Tavanikone C.; Pranamornkith T. Effects of Hot Water Combined with UV-C Treatment on Chinese Kale (Brassica Oleracea Var. Alboglabra) during Storage. J. Food Sci. Agric. Technol. 2019, 5, 172–176.

[ref24] Büchert A. M.; Civello P. M.; Martínez G. A. Effect of Hot Air, UV-C, White Light and Modified Atmosphere Treatments on Expression of Chlorophyll Degrading Genes in Postharvest Broccoli (Brassica Oleracea L.) Florets. Sci. Hortic. (Amsterdam). 2011, 127 (3), 214–219. 10.1016/j.scienta.2010.11.001.

[ref25] Darré M.; Valerga L.; Ortiz Araque L. C.; Lemoine M. L.; Demkura P. V.; Vicente A. R.; Concellón A. Role of UV-B Irradiation Dose and Intensity on Color Retention and Antioxidant Elicitation in Broccoli Florets (Brassica Oleracea Var. Italica). Postharvest Biol. Technol. 2017, 128, 76–82. 10.1016/j.postharvbio.2017.02.003.

[ref26] Liu C.; Han X.; Cai L.; Lu X.; Ying T.; Jiang Z. Postharvest UV-B Irradiation Maintains Sensory Qualities and Enhances Antioxidant Capacity in Tomato Fruit during Storage. Postharvest Biol. Technol. 2011, 59 (3), 232–237. 10.1016/j.postharvbio.2010.09.003.

[ref27] Kasim M. U.; Kasim R. Postharvest UV-B Treatments Increased Fructose Content of Tomato (Solanum Lycopersicon L. Cv. Tayfun F1) Harvested at Different Ripening Stages. Food Sci. Technol. 2015, 35 (4), 742–749. 10.1590/1678-457X.0008.

[ref28] Bramley P. M. Regulation of Carotenoid Formation during Tomato Fruit Ripening and Development. J. Exp. Bot. 2002, 53 (377), 2107–2113. 10.1093/jxb/erf059. 12324534

[ref29] Ruiz-Sola M. Á.; Rodríguez-Concepción M. Carotenoid Biosynthesis in Arabidopsis: A Colorful Pathway. Arab. B. 2012, 10, e0158 10.1199/tab.0158. PMC3350171 22582030

[ref30] Barka E. A.; Kalantari S.; Makhlouf J.; Arul J. Impact of UV-C Irradiation on the Cell Wall-Degrading Enzymes during Ripening of Tomato (Lycopersicon Esculentum L.) Fruit. J. Agric. Food Chem. 2000, 48 (3), 667–671. 10.1021/jf9906174. 10725131

[ref31] Hassenberg K.; Huyskens-Keil S.; Herppich W. B. Impact of Postharvest UV-C and Ozone Treatments on Microbiological Properties of White Asparagus (Asparagus Officinalis L.). J. Appl. Bot. Food Qual. 2012, 85 (2), 174–181.

[ref32] ZHENG J.; LI S.-e; ALI M.; HUANG Q.-h.; ZHNEG X.-l.; PANG L.-j. jiang. Effects of UV-B Treatment on Controlling Lignification and Quality of Bamboo (Phyllostachys Prominens) Shoots without Sheaths during Cold Storage. J. Integr. Agric. 2020, 19 (5), 1387–1395. 10.1016/S2095-3119(20)63170-7.

[ref33] Santos A. L.; Oliveira V.; Baptista I.; Henriques I.; Gomes N. C. M.; Almeida A.; Correia A.; Cunha A. Wavelength Dependence of Biological Damage Induced by UV Radiation on Bacteria. Arch. Microbiol. 2013, 195 (1), 63–74. 10.1007/s00203-012-0847-5. 23090570

[ref34] Cabaj A.; Sommer R.; Schoenen D. Biodosimetry: Model Calculations for u.v. Water Disinfection Devices with Regard to Dose Distributions. Water Res. 1996, 30 (4), 1003–1009. 10.1016/0043-1354(95)00256-1.

[ref35] Song K.; Mohseni M.; Taghipour F. Application of Ultraviolet Light-Emitting Diodes (UV-LEDs) for Water Disinfection: A Review. Water Res. 2016, 94, 341–349. 10.1016/j.watres.2016.03.003. 26971809

[ref36] Huang R.; Chen H. Comparison of Water-Assisted Decontamination Systems of Pulsed Light and Ultraviolet for Salmonella Inactivation on Blueberry, Tomato, and Lettuce. J. Food Sci. 2019, 84 (5), 1145–1150. 10.1111/1750-3841.14510. 31012975

[ref37] Jeong Y. J.; Ha J. W. Combined Treatment of UV-A Radiation and Acetic Acid to Control Foodborne Pathogens on Spinach and Characterization of Their Synergistic Bactericidal Mechanisms. Food Control 2019, 106, 106698 10.1016/j.foodcont.2019.06.024.

[ref38] Woo H. J.; Kang J. H.; Lee C. H.; Song K. Bin. Inactivation of Listeria Monocytogenes, Escherichia Coli O157:H7, and Pre-Existing Bacteria on Spinach by Combined Treatment of Cudrania Tricuspidata Leaf Extract Washing and Ultraviolet-C Irradiation. Food Bioprocess Technol. 2020, 13 (7), 1229–1239. 10.1007/s11947-020-02476-z.

[ref39] Gogo E. O.; Opiyo A. M.; Hassenberg K.; Ulrichs C.; Huyskens-Keil S. Postharvest UV-C Treatment for Extending Shelf Life and Improving Nutritional Quality of African Indigenous Leafy Vegetables. Postharvest Biol. Technol. 2017, 129, 107–117. 10.1016/j.postharvbio.2017.03.019.

[ref40] Mercier J.; Baka M.; Reddy B.; Corcuff R.; Arul J. Shortwave Ultraviolet Irradiation for Control of Decay Caused by Botrytis Cinerea in Bell Pepper: Induced Resistance and Germicidal Effects. J. Am. Soc. Hortic. Sci. 2001, 126 (1), 128–133. 10.21273/JASHS.126.1.128.

[ref41] Artés-Hernández F.; Escalona V. H.; Robles P. A.; Martínez-Hernández G. B.; Artés F. Effect of UV-C Radiation on Quality of Minimally Processed Spinach Leaves. J. Sci. Food Agric. 2009, 89 (3), 414–421. 10.1002/jsfa.3460.

[ref42] Escalona V. H.; Aguayo E.; Martínez-Hernández G. B.; Artés F. UV-C Doses to Reduce Pathogen and Spoilage Bacterial Growth in Vitro and in Baby Spinach. Postharvest Biol. Technol. 2010, 56 (3), 223–231. 10.1016/j.postharvbio.2010.01.008.

[ref43] Cossu A.; Huang K.; Cossu M.; Tikekar R. V.; Nitin N. Fog, Phenolic Acids and UV-A Light Irradiation: A New Antimicrobial Treatment for Decontamination of Fresh Produce. Food Microbiol. 2018, 76, 204–208. 10.1016/j.fm.2018.05.013. 30166142

[ref44] Pattison D. I.; Davies M. J. Actions of Ultraviolet Light on Cellular Structures. EXS. 2006, 96, 131–157. 10.1007/3-7643-7378-4_6. 16383017

[ref45] Guerrero-Beltrán J. A.; Barbosa-Cánovas G. V. Review: Advantages and Limitations on Processing Foods by UV Light. Food Sci. Technol. Int. 2004, 10 (3), 137–147. 10.1177/1082013204044359.

[ref46] Alegria C.; Pinheiro J.; Duthoit M.; Gonçalves E. M.; Moldão-Martins M.; Abreu M. Fresh-Cut Carrot (Cv. Nantes) Quality as Affected by Abiotic Stress (Heat Shock and UV-C Irradiation) Pre-Treatments. LWT - Food Sci. Technol. 2012, 48 (2), 197–203. 10.1016/j.lwt.2012.03.013.

[ref47] Huyskens-Keil S.; Hassenberg K.; Herppich W. B. Impact of Postharvest UV-C and Ozone Treatment on Textural Properties of White Asparagus (Asparagus Offi Cinalis L.). J. Appl. Bot. Food Qual. 2011, 84 (2), 229–234.

[ref48] Kasim M. U.; Kasim R. The Effects of Ultraviolet B (UV-B) Irradiation on Color Quality and Decay Rate of Capia Pepper during Postharvest Storage. Food Sci. Technol. 2018, 38 (2), 363–368. 10.1590/1678-457x.05817.

[ref49] Gogo E. O.; Förster N.; Dannehl D.; Frommherz L.; Trierweiler B.; Opiyo A. M.; Ulrichs C.; Huyskens-Keil S. Postharvest UV-C Application to Improve Health Promoting Secondary Plant Compound Pattern in Vegetable Amaranth. Innov. Food Sci. Emerg. Technol. 2018, 45, 426–437. 10.1016/j.ifset.2018.01.002.

[ref50] Wu J.; Liu W.; Yuan L.; Guan W. Q.; Brennan C. S.; Zhang Y. Y.; Zhang J.; Wang Z. D. The Influence of Postharvest UV-C Treatment on Anthocyanin Biosynthesis in Fresh-Cut Red Cabbage. Sci. Rep. 2017, 7 (1), 1–11. 10.1038/s41598-017-04778-3. 28701702 PMC5507880

[ref51] Liu C.; Zheng H.; Sheng K.; Liu W.; Zheng L. Effects of Postharvest UV-C Irradiation on Phenolic Acids, Flavonoids, and Key Phenylpropanoid Pathway Genes in Tomato Fruit. Sci. Hortic. (Amsterdam). 2018, 241 (June), 107–114. 10.1016/j.scienta.2018.06.075.

[ref52] Castagna A.; Dall”Asta C.; Chiavaro E.; Galaverna G.; Ranieri A. Effect of Post-Harvest UV-B Irradiation on Polyphenol Profile and Antioxidant Activity in Flesh and Peel of Tomato Fruits. Food Bioprocess Technol. 2014, 7 (8), 2241–2250. 10.1007/s11947-013-1214-5.

[ref53] Formica-Oliveira A. C.; Martínez-Hernández G. B.; Díaz-López V.; Artés F.; Artés-Hernández F. Use of Postharvest UV-B and UV-C Radiation Treatments to Revalorize Broccoli Byproducts and Edible Florets. Innov. Food Sci. Emerg. Technol. 2017, 43, 77–83. 10.1016/j.ifset.2017.07.036.

[ref54] Yoon H. I.; Kim D.; Son J. E. Spatial and Temporal Bioactive Compound Contents and Chlorophyll Fluorescence of Kale (Brassica Oleracea L.) Under UV-B Exposure Near Harvest Time in Controlled Environments. Photochem. Photobiol. 2020, 96 (4), 845–852. 10.1111/php.13237. 32104924

[ref55] Du W. X.; Avena-Bustillos R. J.; Breksa A. P.; McHugh T. H. Effect of UV-B Light and Different Cutting Styles on Antioxidant Enhancement of Commercial Fresh-Cut Carrot Products. Food Chem. 2012, 134 (4), 1862 10.1016/j.foodchem.2012.03.097. 23442631

[ref56] Zhang J.; Yuan L.; Liu W.; Lin Q.; Wang Z.; Guan W. Effects of UV-C on Antioxidant Capacity, Antioxidant Enzyme Activity and Colour of Fresh-Cut Red Cabbage during Storage. Int. J. Food Sci. Technol. 2017, 52 (3), 626–634. 10.1111/ijfs.13315.

[ref57] Romano R.; Pizzolongo F.; De Luca L.; Cozzolino E.; Rippa M.; Ottaiano L.; Mormile P.; Mori M.; Di Mola I. Bioactive Compounds and Antioxidant Properties of Wild Rocket (Diplotaxis Tenuifolia L.) Grown under Different Plastic Films and with Different UV-B Radiation Postharvest Treatments. Foods 2022, 11 (24), 4093 10.3390/foods11244093. 36553834 PMC9778044

[ref58] Gallie D. R. L-Ascorbic Acid: A Multifunctional Molecule Supporting Plant Growth and Development. Scientifica (Cairo). 2013, 2013, 1–24. 10.1155/2013/795964. PMC3820358 24278786

[ref59] Falk J.; Munné-Bosch S. Tocochromanol Functions in Plants: Antioxidation and Beyond. Journal of Experimental Botany 2010, 61, 1549–1566. 10.1093/jxb/erq030. 20385544

[ref60] Liao C.; Liu X.; Gao A.; Zhao A.; Hu J.; Li B. Maintaining Postharvest Qualities of Three Leaf Vegetables to Enhance Their Shelf Lives by Multiple Ultraviolet-C Treatment. LWT - Food Sci. Technol. 2016, 73, 1–5. 10.1016/j.lwt.2016.05.029.

[ref61] Andrade Cuvi M. J.; Vicente A. R.; Concellón A.; Chaves A. R. Changes in Red Pepper Antioxidants as Affected by UV-C Treatments and Storage at Chilling Temperatures. LWT - Food Sci. Technol. 2011, 44 (7), 1666–1671. 10.1016/j.lwt.2011.01.027.

[ref62] LIU C.-h.; CAI L.-y.; LU X.-y.; HAN X.-x.; YING T.-j. Effect of Postharvest UV-C Irradiation on Phenolic Compound Content and Antioxidant Activity of Tomato Fruit During Storage. J. Integr. Agric. 2012, 11 (1), 159–165. 10.1016/S1671-2927(12)60794-9.

[ref63] Kanazawa K.; Hashimoto T.; Yoshida S.; Sungwon P.; Fukuda S. Short Photoirradiation Induces Flavonoid Synthesis and Increases Its Production in Postharvest Vegetables. J. Agric. Food Chem. 2012, 60 (17), 4359–4368. 10.1021/jf300107s. 22506664

[ref64] Castagna A.; Chiavaro E.; Dall”Asta C.; Rinaldi M.; Galaverna G.; Ranieri A. Effect of Postharvest UV-B Irradiation on Nutraceutical Quality and Physical Properties of Tomato Fruits. Food Chem. 2013, 137 (1–4), 151–158. 10.1016/j.foodchem.2012.09.095. 23200003

[ref65] Dai J.; Mumper R. J. Plant Phenolics: Extraction, Analysis and Their Antioxidant and Anticancer Properties. Molecules. 2010, 15, 7313–7352. 10.3390/molecules15107313. 20966876 PMC6259146

[ref66] Grassmann J.; Hippeli S.; Elstner E. F. Plant”s Defence and Its Benefits for Animals and Medicine: Role of Phenolics and Terpenoids in Avoiding Oxygen Stress. In Plant Physiology and Biochemistry 2002, 40, 471–478. 10.1016/S0981-9428(02)01395-5.

[ref67] Mercier J.; Arul J.; Ponnampalam R.; Boulet M. Induction of 6-Methoxymellein and Resistance to Storage Pathogens in Carrot Slices by UV-C. J. Phytopathol. 1993, 137 (1), 44–54. 10.1111/j.1439-0434.1993.tb01324.x.

[ref68] Ojaghian M. R.; Zhang J. Z.; Xie G. L.; Wang Q.; Li X. L.; Guo D. P. Efficacy of UV-C Radiation in Inducing Systemic Acquired Resistance against Storage Carrot Rot Caused by Sclerotinia Sclerotiorum. Postharvest Biol. Technol. 2017, 130, 94–102. 10.1016/j.postharvbio.2017.04.009.

[ref69] Rybarczyk-Plonska A.; Wold A. B.; Bengtsson G. B.; Borge G. I. A.; Hansen M. K.; Hagen S. F. Flavonols in Broccoli (Brassica Oleracea L. Var. Italica) Flower Buds as Affected by Postharvest Temperature and Radiation Treatments. Postharvest Biol. Technol. 2016, 116, 105–114. 10.1016/j.postharvbio.2015.12.023.

[ref70] Kowalski A.; Agati G.; Grzegorzewska M.; Kosson R.; Kusznierewicz B.; Chmiel T.; Bartoszek A.; Tuccio L.; Grifoni D.; Vågen I. M.; Kaniszewski S. Valorization of Waste Cabbage Leaves by Postharvest Photochemical Treatments Monitored with a Non-Destructive Fluorescence-Based Sensor. J. Photochem. Photobiol. B Biol. 2021, 222 (July), 112263 10.1016/j.jphotobiol.2021.112263. 34339994

[ref71] Urban L.; Charles F.; de Miranda M. R. A.; Aarrouf J. Understanding the Physiological Effects of UV-C Light and Exploiting Its Agronomic Potential before and after Harvest. Plant Physiol. Biochem. 2016, 105, 1–11. 10.1016/j.plaphy.2016.04.004. 27064192

[ref72] Ribeiro C.; Canada J.; Alvarenga B. Prospects of UV Radiation for Application in Postharvest Technology. Emirates J. Food Agric. 2012, 24 (6), 586–597. 10.9755/ejfa.v24i6.14677.

[ref73] Turtoi M. Ultraviolet Light Treatment of Fresh Fruits and Vegetables Surface: A Review. J. Agroaliment. Process. Technol. 2013, 19 (3), 325–337.

[ref74] Liu R.; Zhou J. L.; Wilding A. Simultaneous Determination of Endocrine Disrupting Phenolic Compounds and Steroids in Water by Solid-Phase Extraction-Gas Chromatography-Mass Spectrometry. J. Chromatogr. A 2004, 1022 (1–2), 179–189. 10.1016/j.chroma.2003.09.035. 14753785

[ref75] Harbaum-Piayda B.; Palani K.; Schwarz K. Influence of Postharvest UV-B Treatment and Fermentation on Secondary Plant Compounds in White Cabbage Leaves. Food Chem. 2016, 197, 47–56. 10.1016/j.foodchem.2015.10.065. 26616923

[ref76] Atkinson N. J.; Dew T. P.; Orfila C.; Urwin P. E. Influence of Combined Biotic and Abiotic Stress on Nutritional Quality Parameters in Tomato (Solanum Lycopersicum). J. Agric. Food Chem. 2011, 59 (17), 9673–9682. 10.1021/jf202081t. 21830786

[ref77] Gautier H.; Diakou-Verdin V.; Bénard C.; Reich M.; Buret M.; Bourgaud F.; Poëssel J. L.; Caris-Veyrat C.; Génard M. How Does Tomato Quality (Sugar, Acid, and Nutritional Quality) Vary with Ripening Stage, Temperature, and Irradiance?. J. Agric. Food Chem. 2008, 56 (4), 1241–1250. 10.1021/jf072196t. 18237131

[ref78] Sonntag F.; Bunzel D.; Kulling S.; Porath I.; Pach F.; Pawelzik E.; Smit I.; Naumann M. Effect of Potassium Fertilization on the Concentration of Antioxidants in Two Cocktail Tomato Cultivars. J. Appl. Bot. Food Qual. 2020, 93, 34–43. 10.5073/JABFQ.2020.093.005.

[ref79] Egea I.; Barsan C.; Bian W.; Purgatto E.; Latché A.; Chervin C.; Bouzayen M.; Pech J. C. Chromoplast Differentiation: Current Status and Perspectives. Plant Cell Physiol. 2010, 51 (10), 1601–1611. 10.1093/pcp/pcq136. 20801922

[ref80] Aiamla-or S.; Nakajima T.; Shigyo M.; Yamauchi N. Pheophytinase Activity and Gene Expression of Chlorophyll-Degrading Enzymes Relating to UV-B Treatment in Postharvest Broccoli (Brassica Oleracea L. Italica Group) Florets. Postharvest Biol. Technol. 2012, 63 (1), 60–66. 10.1016/j.postharvbio.2011.08.003.

[ref81] Sønderby I. E.; Geu-Flores F.; Halkier B. A. Biosynthesis of Glucosinolates - Gene Discovery and Beyond. Trends Plant Sci. 2010, 15 (5), 283–290. 10.1016/j.tplants.2010.02.005. 20303821

[ref82] Blažević I.; Montaut S.; Burčul F.; Olsen C. E.; Burow M.; Rollin P.; Agerbirk N. Glucosinolate Structural Diversity, Identification, Chemical Synthesis and Metabolism in Plants. Phytochemistry 2020, 169, 112100 10.1016/j.phytochem.2019.112100. 31771793

[ref83] Baenas N.; Cartea M. E.; Moreno D. A.; Tortosa M.; Francisco M. Processing and Cooking Effects on Glucosinolates and Their Derivatives. In Glucosinolates: Properties, Recovery, and Applications; Elsevier, 2020; pp 181–212. 10.1016/B978-0-12-816493-8.00006-8.

[ref84] Kissen R.; Eberl F.; Winge P.; Uleberg E.; Martinussen I.; Bones A. M. Effect of Growth Temperature on Glucosinolate Profiles in Arabidopsis Thaliana Accessions. Phytochemistry 2016, 130, 106–118. 10.1016/j.phytochem.2016.06.003. 27319377

[ref85] Duarte-Sierra A.; Nadeau F.; Angers P.; Michaud D.; Arul J. UV-C Hormesis in Broccoli Florets: Preservation, Phyto-Compounds and Gene Expression. Postharvest Biol. Technol. 2019, 157 (July), 110965 10.1016/j.postharvbio.2019.110965.

[ref86] Martínez-Zamora L.; Castillejo N.; Artés-Hernández F. Postharvest UV-B and UV-C Radiation Enhanced the Biosynthesis of Glucosinolates and Isothiocyanates in Brassicaceae Sprouts. Postharvest Biol. Technol. 2021, 181, 111650 10.1016/j.postharvbio.2021.111650.

[ref87] Barba-Espin G.; Glied-Olsen S.; Dzhanfezova T.; Joernsgaard B.; Lütken H.; Müller R. Preharvest Application of Ethephon and Postharvest UV-B Radiation Improve Quality Traits of Beetroot (Beta Vulgaris L. Ssp. Vulgaris) as Source of Colourant. BMC Plant Biol. 2018, 18 (1), 316 10.1186/s12870-018-1556-2. 30509181 PMC6276243

[ref88] Nakashima T.; Araki T.; Ueno O. Photoprotective Function of Betacyanin in Leaves of Amaranthus Cruentus L. under Water Stress. Photosynthetica 2011, 49 (4), 497–506. 10.1007/s11099-011-0062-7.

[ref89] Jain G.; Gould K. S. Are Betalain Pigments the Functional Homologues of Anthocyanins in Plants?. Environ. Exp. Bot. 2015, 119, 48–53. 10.1016/j.envexpbot.2015.06.002.

[ref90] Falguera V.; Pagán J.; Garza S.; Garvín A.; Ibarz A. Ultraviolet Processing of Liquid Food: A Review. Part 2: Effects on Microorganisms and on Food Components and Properties. Food Research International. 2011, 44, 1580–1588. 10.1016/j.foodres.2011.03.025.

